# Characterization of Nanoparticles in Ethanolic Suspension Using Single Particle Inductively Coupled Plasma Mass Spectrometry: Application for Cementitious Systems

**DOI:** 10.1021/acsomega.4c01196

**Published:** 2024-07-02

**Authors:** Steffen Hellmann, Teba Gil-Díaz, Marcus Böhm, Dirk Merten, Sylvain Grangeon, Fabienne Warmont, Sophie Unbehau, Thomas Sowoidnich, Thorsten Schäfer

**Affiliations:** † Friedrich Schiller University Jena, Institute of Geosciences, Applied Geology, Burgweg 11, 07749 Jena, Germany; ‡ International Max Planck Research School for Global Biogeochemical Cycles, Max Planck Institute for Biogeochemistry, Department of Biogeochemical Processes, Hans-Knöll-Straße 10, 07745 Jena, Germany; § BRGM, 3 Avenue Claude-Guillemin, F-45060 Orléans, France; ∥ ICMN, 1B, rue de la Férollerie CS40059, F-45071 Cedex 2 Orléans, France; ⊥ Bauhaus-Universität Weimar, Institute for Building Materials, Coudraystr. 11, 99423 Weimar, Germany

## Abstract

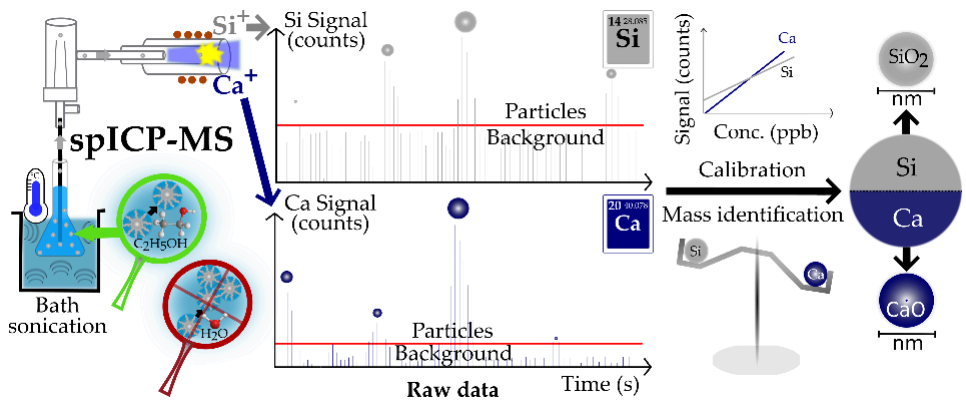

Single particle inductively coupled plasma mass spectrometry (spICP-MS) is a well-established technique to characterize the size, particle number concentration (PNC), and elemental composition of engineered nanoparticles (NPs) and colloids in aqueous suspensions. However, a method capable of directly analyzing water-sensitive or highly reactive NPs in alcoholic suspension has not been reported yet. Here, we present a novel spICP-MS method for characterizing the main cement hydration product, i.e., calcium-silicate-hydrate (C-S-H) NPs, in ethanolic suspensions, responsible for cement strength. The method viability was tested on a wide range of NP compositions and sizes (i.e., from Au, SiO_2_, and Fe_3_O_4_ NP certified reference materials (CRMs) to synthetic C-S-H phases with known Ca/Si ratios and industrial cement hardening accelerators, X-Seed 100/500). Method validation includes comparisons to nanoparticle tracking analysis (NTA) and transmission/scanning electron microscopy (TEM/SEM). Results show that size distributions from spICP-MS were in good agreement with TEM and NTA for CRMs ≥ 51 nm and the synthetic C-S-H phases. The X-Seed samples showed significant differences in NP sizes depending on the elemental composition, i.e. CaO and SiO_2_ NPs were bigger than Al_2_O_3_ NPs. PNC via spICP-MS was successfully validated with an accuracy of 1 order of magnitude for CRMs and C-S-H phases. The spICP-MS Ca/Si ratios matched known ratios from synthetic C-S-H phases (0.6, 0.8, and 1.0). Overall, our method is applicable for the direct and element-specific quantification of fast nucleation and/or mineral formation processes characterizing NPs (ca. 50–1000 nm) in alcoholic suspensions.

## 1 Introduction

Nanoparticles (NPs) are ubiquitous in environmental matrices such as (sub)surface waters, soils, biota, and the atmosphere even though they were historically overlooked when describing and characterizing environmental systems. NPs pose a potential danger to human and environmental health because they are easily taken up by biota due to their small size.^1^ However, NPs are commonly used in industry, for example, as additives in food, pharmaceutics, or cosmetics. For instance, the main cement hydration product is a nanocrystalline phase, termed calcium-silicate-hydrate (C-S-H in cement nomenclature). Cements are also often artificially enriched in Fe_2_O_3_, TiO_2_, Al_2_O_3_, SiO_2_ NPs, or C-S-H additives such as “Celitement”^2^ to improve properties such as strength, water permeability, abrasion resistance, and pore structure.^3,4^ SiO_2_ NPs are of special importance in order to achieve high quality and strong concrete during cement hydration, acting as trigger and nucleus to the onset of stronger bond forming products, the C-S-H phases.^5^ Investigating C-S-H size and composition is, however, challenging in aqueous matrices and virtually impossible in pure water due to their high solubility. The use of bipolar organic solvents such as alcoholic matrices allow circumvention of this difficulty. In previous studies, isopropyl alcohol was successfully used to resuspend cementitious NPs and stop the hydration process by removing water.^6^ However, an efficient method is required to further characterize these NPs in such alcoholic matrices without elaborate sample preparation.

Until now, the elemental composition of suspensions of inorganic NPs was usually studied using bulk ICP-MS after acid digestion. For this, a drying step of NP suspensions was necessary, potentially resulting in crystallization artifacts. Bulk ICP-MS produced a result in which particle composition and concentration were averaged, and hence, only an average NP composition and concentration could be determined. However, single particle inductively coupled plasma mass spectrometry (spICP-MS) is a promising method to characterize NPs and colloids (20–5000 nm)^7^ in suspension with respect to their size, particle number concentration (PNC) and elemental composition. In the past decades, it has become a well-established method for the characterization of engineered, inorganic NPs and colloids in aqueous suspensions. The feasibility study of this technique was to our knowledge first reported by Degueldre and Favarger, who investigated several inorganic colloids (150–400 nm) in aqueous suspensions.^8^ The method was applied for several particle types such as nano- and microplastics, natural particles such as clay and biological cells (known as single cell ICP-MS).^9^ Until now, ca. 880 publications (Scopus, August 2023) focused on the characterization of NPs via spICP-MS, most commonly containing Au,^10,11^ Ag,^8,10−12^ TiO_2_,^11,13^ ZnO,^10,11^ CeO_2_,^11^ and SiO_2_,^14^ have been released.^9^

Compared to other techniques such as nanoparticle tracking analysis (NTA), dynamic light scattering (DLS), or asymmetrical flow field flow fractionation (AF4), which mainly focus on particle size and/or number concentration, spICP-MS has the advantage of providing additional elemental composition. Complementary information is received by scanning and transmission electron microscopy (SEM and TEM, respectively) which are also capable of providing NP sizes and, if equipped with energy-dispersive X-ray analysis (EDX), chemical composition. Differences are that NPs can directly be analyzed in suspension using spICP-MS, while SEM and TEM require dry NPs, which is often problematic in terms of agglomeration. Nevertheless, SEM and TEM provide information about NP morphologies and can therefore not be fully replaced by spICP-MS. However, spICP-MS is more powerful in providing simultaneously and independently the PNC and element-specific masses and sizes for each and many individual particles. Another important benefit of spICP-MS is the high sample throughput,^15^ requiring only low particle concentrations of around 10^5^ NP mL^–1^.^16^ Pace et al. reported that a minimum concentration of 10^3^ NP mL^–1^ is statistically enough to run a spICP-MS measurement, underlining the uniqueness and high sensitivity of the technique.^12^ The sample throughput is limited by the transport efficiency (TE), that is the percentage of NPs which eventually enter the plasma. Usually, when applying spICP-MS for aqueous matrices, Scott double pass or cyclonic spray chambers are used even though they result in low TE (between 2 and 10%).^9^ Alcohols such as ethanol have a lower density and higher steam pressure compared to water, resulting in more aerosol arriving in the plasma leading to a higher TE. When analyzing heterogeneous particle suspensions, this avoids the exclusion of entire particle groups.

Nevertheless, spICP-MS analysis remains challenging. For instance, while the spICP-MS analysis of well-defined engineered NPs is a well-established procedure as shape, size, density, and mostly monoanalyte-containing NP compositions are known, the investigation of unknown multianalyte and/or natural NPs is much more complex. In fact, analyses of unknown NPs involve some assumptions such as spherical shapes for the aforementioned parameters in order to calculate particle sizes. This means that the results from spICP-MS will always be biased for particles with shapes different than spherical geometries, as spICP-MS cannot provide information about individual particle shapes to adjust size calculations systematically assuming other than spherical particles. Another challenge in spICP-MS is that, depending on the particle type, matrix, and corresponding zeta potentials, NPs might tend to agglomerate in suspension resulting in an underestimated PNC and overestimated sizes. To prevent this, particle stabilization agents such as citrate^9^ can be added. However, this is not always possible as some NPs might partly react and dissolve in the presence of citrate. Alternatively, the sample can be sonicated (bath or probe) prior to analysis. However, extensive sonication leads to overheating and might also result in partial particle dissolution.^16,17^ In our work, however, we used a temperature-controlled bath system to overcome overheating issues during sonication. The last, and one of the greatest, challenge of the spICP-MS technique during elemental quantification is the accurate separation between ionic or background signals and those corresponding to small particles, particularly for unknown multianalyte particle suspensions. To tackle this at the postprocessing level, several approaches defining the particle threshold during data treatment are often based on iterative methods that average the whole data set and collect only the data that are 3 or 5 times above the standard deviation (SD) from the average of the entire data set.^18,19^ This approach was often combined with signal deconvolution methods which commonly use Poisson and (polya)Gaussian fits, particularly useful when the ionic signal or background distribution overlaps with the particle distribution.^20^ One of the factors contributing to high background signals in ICP-MS are spectral interferences, particularly challenging for elements with *m*/*z* ≤ 80.^9^ This is the case for the three main elements of interest in cementitious NPs: Al, Ca, and Si. Of these, Si also shows a low signal-to-noise ratio mainly caused by the high background signal from Si-containing glass components within the ICP-MS. Therefore, NP quantification from Si signals is critical for small NPs. Finally, when using dwell times in the μs range, a peak integration approach is necessary as multiple signals are generated within one particle event. A typical particle event duration is, depending on the element and particle size, between 0.4 and 0.9 ms.^21^

Little attention has been paid to the development of spICP-MS methods for matrices other than water. To our knowledge, only a few applications of NP investigation using spICP-MS in organic media have been published until now.^22−26^ None of these include alcoholic matrices, highlighting the need for further research on new methods, especially for highly reactive, water-sensitive particles. Past organic solvents used for spICP-MS were o-xylene,^22,23^ toluene,^24,26^ tetrahydrofuran,^25^ mesitylene,^26^ and dodecanethiol.^26^ In this study, we present an innovative spICP-MS method for quantifying the physical properties of NPs and colloids in pure ethanol, namely, NP size distributions and the order of magnitude in PNC differentiated by elemental content and NP composition. Specifically, we apply and validate our method for certified reference materials (CRMs, containing Au, SiO_2_ ,and Fe_3_O_4_), showing its application for C-S-H phases with known Ca/Si ratios and for unknown industrial cement hardening accelerators (X-Seed 100 and 500). This approach opens up a new field of application for all kinds of fast nucleation and/or hydration systems where reactive, water-sensitive NPs change fast over time. With our approach, we slow down the NP reaction kinetics, while assuring an analytical procedure in a comparably cheap, easily available, and low-(eco)toxicity ethanol matrix.

## Experimental Section

### Material and Reagents

All ionic standards and particle suspensions were prepared in pure ethanol (absolute for analysis, Merck, Darmstadt, Germany). Ionic calibrations (0–50 μg L^–1^) were diluted in quartz glass volumetric flasks to minimize ionic background (except Si). Glass labware was used throughout this study to increase bath sonication efficiency for NP deagglomeration prior to spICP-MS. All ionic standards are listed in the Supporting Information in Table S1, and the NP reference materials are listed in Table S2.

### Sample Characteristics and Preparation

There are several definitions in the literature concerning the term nanomaterials/NPs. The most typical one includes only particles showing 1 to 100 nm sizes in one or more external dimensions.^27^ Another definition, particularly used in chemical engineering, comprises particle sizes between 1 and 1000 nm. In our work, the particles ranged between 10 and 1000 nm, including more than 80% of the samples below or equal to the 100 nm threshold. Therefore, we have used the term NPs for all our samples in this manuscript. Silica-shelled Au nanospheres (20, 50, and 100 nm), aminated SiO_2_ nanospheres (50, 100, 300, and 1000 nm), all certified in ethanol matrix, and PVP surface treated solid Au (10, 50, 100 nm) and Fe_3_O_4_ (20 nm in 2 mM aqueous citrate) NP reference materials were analyzed for method validation. Certified values are shown in Table S2. C-S-H (pH 10.0–12.5) materials were synthesized at four target molar Ca/Si ratios of 0.6, 0.8, 1.0, and 1.2 (hereafter referred to as “C-S-H *X*”, where *X* is the target Ca/Si) (Table S3) and investigated for method validation. Details on the synthesis can be found elsewhere.^28^ Additionally, two industrial products (X-Seed 100 and X-Seed 500; both from Master Builders Solutions Deutschland GmbH, Germany) were investigated to further verify the spICP-MS method. The X-Seed admixtures are cement hardening accelerators consisting of crystalline NPs, i.e., C-S-H and other supplements such as reaction educts or superplasticizers. X-Seed 100 NPs are originally available as suspensions in an aqueous solution (22 ± 1.0% solid content) at pH 11.^29^ X-Seed 500 is originally in powder form.

NP CRMs and C-S-H (synthetical, industrial) samples were always diluted in ethanol to suspensions with final nominal concentrations of ∼10^5^ NPs mL^–1^. Prior to spICP-MS investigation, all suspensions were sonicated (SONOCOOL 255.2, Bandelin, Berlin, Germany) for ≥15 min at 20 °C to deagglomerate NPs.

### Digestions and Bulk ICP-OES of X-Seed 100/500

Given the fact that the X-Seed materials are not CRMs, we performed additional measurements in order to obtain their elemental compositions and Ca/Si ratios for method validation of the spICP-MS data. For the case of the X-Seed 100, the NPs were extracted from the original aqueous suspension via centrifugation, discarding the supernatant, resuspending the NPs in ethanol, and repeating this step one more time to remove leftover water. For the case of the X-Seed 100, we used both the original suspension (without washing) and an aliquot from the ethanol resuspension after centrifugation for acid digestions. Both samples were dried for ≥24 h at 40 °C prior to digestion. The X-Seed 500 is originally a dry powder and was used in its original state without drying (water content <1%). Overall, 50–100 mg (not washed, later referred to as “Original”) vs 2–3 mg (ethanol-washed, later referred to as “small weight”) of the dry powders were digested via microwave (Mars 5 Xpress, CEM, Kamp-Lintfort, Germany) using aqua regia. 35–100 mg X-Seed 100 and 500 was additionally digested via conventional pressure digestion (DAS, PicoTrace, Bovenden, Germany) using hydrofluoric (HF) acid total digestion for comparison. All solutions were measured using a 725ES ICP-OES as bulk analysis (Agilent Technologies, Waldbronn, Germany).

### Additional Validation Techniques

Transmission electron microscopy (TEM) was performed using a CM-20 (Philips, Hamburg, Germany) operated at 200 kV for the C-S-H samples. Selected area electron diffraction (SAED) was used to confirm that analyzed samples had C-S-H structure. For TEM, aqueous suspensions were filtered (<0.2 μm, cellulose acetate (CA), Sartorius AG, Göttingen, Germany) and rinsed with ethanol to remove leftover pore water, to retain the NPs. NPs were then resuspended in ethanol. A drop of the obtained suspension was deposited on a lacey carbon film loaded on a Cu grid.

Scanning electron microscopy (SEM) was performed using a Zeiss Ultra Plus SEM (Zeiss AG, Jena, Germany) operated at 20.0 kV equipped with an electron dispersive X-ray (EDX) detector (XFlash 6|30, Bruker, Billerica, USA) to provide an additional estimate on elemental particle compositions (Ca/Si ratios) for the X-Seed samples (100 and 500). EDX was done at three different locations of compacted NPs in three to five replicates each to ensure that the 1 μm X-ray beam records only particles not background. Prior to analysis, the X-Seed 100 aqueous suspension was dried at 40 °C; X-Seed 500 was originally a powder. Both solids were attached to a carbon pad and coated with a thin carbon layer. Additionally, particle sizes and number concentrations were estimated in higher dilutions via SEM operated at 10.0 kV. For that, each X-Seed sample was diluted in ethanol 10^5^, 10^6^, and 10^7^ times, and the particle suspension was centrifuged (Sorvall LYNX 4000, Thermo Fisher Scientific, Schwerte, Germany) for 8 h at 20,000*g*, 17–18 °C, onto Si wafers laying on resin filled (Araldite 2020 (XW 396/XW 397), Huntsman Advanced Materials GmbH, Basel, Switzerland) 50 mL polypropylene-copolymer centrifuge tubes. This approach avoided saturation and allowed having distinctive particles on the Si-wafer, from which we could count the number of particles in each suspension. This information, together with the estimated total dilution factors and suspension volumes from gravimetric measurements before centrifugation, provided the concentration of particles for each sample. Further information can be found in Tables S4 and S5 and eq S1. SEM and TEM sizes were estimated by measuring and averaging the smallest and largest diameter of each particle when possible, or by measuring one direction when particles were overlapping.

X-ray fluorescence (XRF) was performed with a wavelength dispersive X-ray spectrometer (S8 Tiger, Bruker, Germany) under vacuum conditions to analyze the chemical composition of X-Seed 500. The sample was first annealed (1000 °C, 1 h) to determine the loss on ignition and then analyzed as a fused bead. One gram of the preannealed sample material was mixed with 8 g of flux, prepared using Spectromelt A12 (66% Li_2_B_4_O_7_/34% LiBO_2_), and melted in an automatic electric furnace (xrFuse 2, XRF scientific, Australia) with Pt/Rh crucibles.

Nanoparticle tracking analysis (NTA) (NanoSight NS300, Malvern Panalytical Ltd., Malvern, United Kingdom) was used to estimate hydrodynamic particle diameters and particle number concentrations (PNCs) for all samples. Samples were diluted in ethanol to final concentrations between 5 × 10^6^ and 5 × 10^9^ NP mL^–1^. The camera level, shutter, and gain were individually optimized for each sample. A 100 nm Polystyrene Latex standard NTA4088 (Malvern Panalytical Ltd.) was daily tested to ensure measurement quality. All other NTA settings can be found in Table S6.

### spICP-MS Instrumentation

All spICP-MS measurements were performed using an 8900 ICP-MS/MS (Agilent Technologies) equipped with a set for organic solvents consisting of a concentric MicroMist nebulizer, a Scott double pass spray chamber, and a torch with a 1 mm diameter injection tube, all made of quartz glass from Agilent Technologies. The sampler (Pt-based) and skimmer cone (Pt/Ni-based) were used in combination with a brass skimmer base equipped with x-lenses (Agilent Technologies), necessary when using organic solvents such as ethanol. Additionally, a low gas stream of Ar/O_2_ was used to oxidize carbon and therefore prevent carbon deposition on the cones. The optimized spICP-MS settings are listed in Table 1.

**Table 1:** Optimized spICP-MS Settings

parameter	value
RF power (W)	1600
sample depth (mm)	9
nebulizer gas (L min^–1^)	0.50–0.70
make-up gas (L min^–1^)	0
Ar/O_2_ (80%/20%) gas (L min^–1^)	0.135
nebulizer pump (rps)	0.10
spray chamber temp. (°C)	–5
dwell time (s)	0.0001
total acquisition time (s)	40–60

The sample was introduced self-aspirated, without a pump, in order to provide a consistent inlet flow. This flow was monitored gravimetrically on a daily basis, varying between 150 and 250 μL min^–1^ mainly depending on the optimized nebulizer gas flow. A peristaltic pump was used to empty the waste from the spray chamber. The instrument was daily tuned using a multielement tune solution (10 μg L^–1^ Ce, Co, Li, Tl, and Y in ethanol) by maximizing the sensitivity, while keeping the oxides ratio (^140^Ce^16^O^+^/^140^Ce^+^) and doubly charged species (^140^Ce^2+^/^140^Ce^+^) below 3 and 5%, respectively. The transport efficiency (TE), describing how many NPs from the particle suspension made it to the plasma, was determined daily using the waste collection method.^30^ For this, a 15 mL centrifuge tube with a fine hole in the cap was used to collect waste flow that was then gravimetrically determined. Cell gas flow was chosen and optimized based on the best signal/noise ratio and background equivalent concentration (BEC) (Supporting Information, Figure S1). Major interferences originated from the carbon-rich ethanol matrix. In particular, ^24^Mg^+^ was interfered by ^12^C^12^C^+^ and ^27^Al^+^ by ^13^C^14^N^+^ and ^12^C^15^N^+^.^31^ However, these interferences could be minimized using ammonia cell gas (Table 2).

**Table 2:** Chosen Isotopes, Cell Gas, and Flow for spICP-MS

isotope	gas	gas flow (mL min^–1^)
^24^Mg^+^ → ^24^Mg^+^	NH_3_/He (90%/10%)	2.0
	He (100%)	1.0
^27^Al^+^ → ^27^Al^+^	NH_3_/He (90%/10%)	2.0
	He (100%)	1.0
^28^Si^+^ → ^28^Si^+^	Mixed: H_2_ & O_2_	4.0 & 0.15
^32^S^+^ → ^32^S^16^O^+^	O_2_	0.45
^40^Ca^+^ → ^40^Ca^+^	H_2_	6.5
^56^Fe^+^ → ^56^Fe^+^	mixed H_2_ & O_2_	4.0 & 0.15
^197^Au^+^	no gas	

### Data Treatment for spICP-MS

An in-house Python code was used for processing all spICP-MS data. Briefly, an iterative method (Gaussian approach) was applied based on averaging the whole data set and extracting all data points higher than the mean (μ) + *k* × standard deviation (SD).^18,32,33^ Factor *k* was individually set for each sample based on the background level. The final value was used as a threshold to identify particle events. For low background elements (<50,000 counts per second (cps) background) such as Au, instead of the Gaussian approach, a Poisson approach was more suitable and thus used to identify the particle detection threshold.^34^ Second, a peak finding algorithm was applied to identify contiguous particle events above the particle detection threshold and sum up individual data points which were associated with one particle. The extracted particle signals were then used to calculate the corresponding masses and sizes of each analyte.^35^ Finally, outliers which had greater than μ + 3SD from all particle events were removed. Size limits of detections (LOD_size_) were individually calculated as shown in the Supporting Information (eqs S2 and S3).

## Results and Discussion

### Sample Characterization

In the following subsections, we discuss the morphology of the four C-S-H phases which were analyzed by TEM and those of the two industrial cement hardening accelerators X-Seed 100 and 500 analyzed by SEM.

#### TEM Observations: C-S-H Morphology

**Figure 1:**
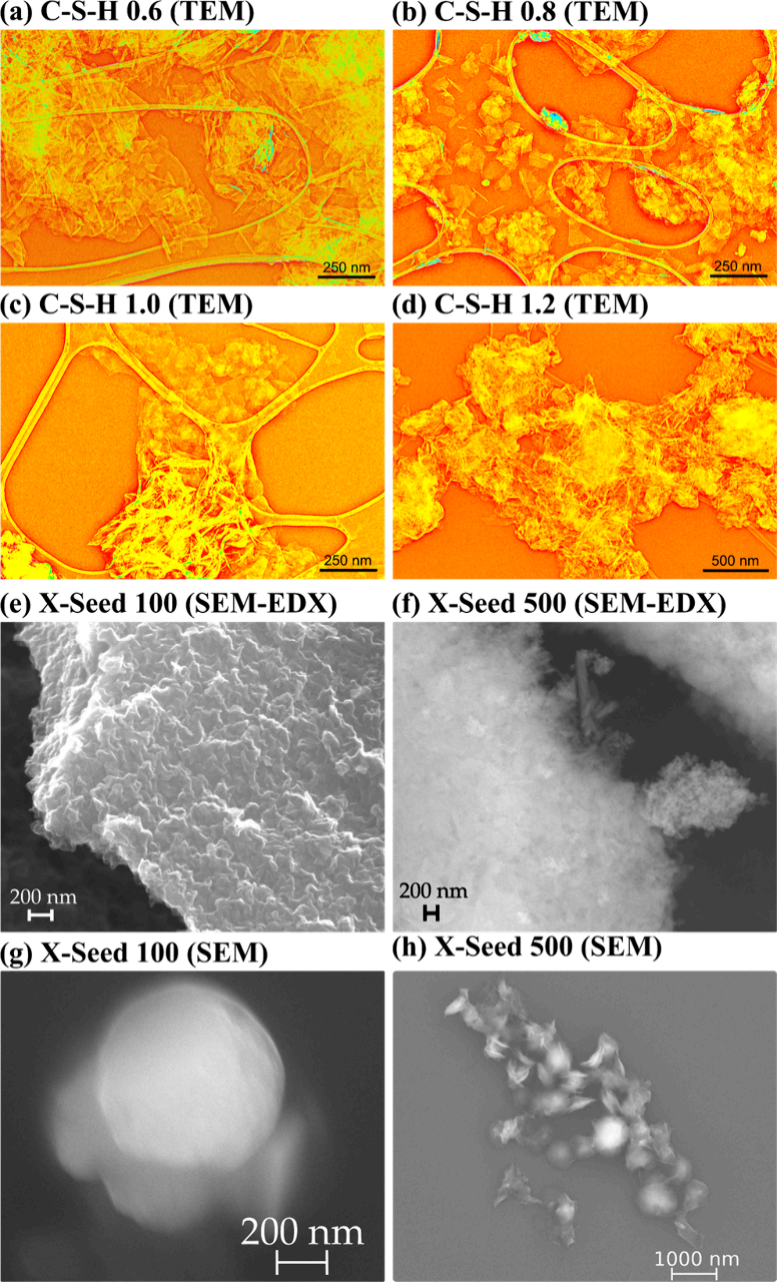
Particle characterization of C-S-H via TEM (in orange) for (a) C-S-H 0.6, (b) C-S-H 0.8, (c) C-S-H 1.0, (d) C-S-H 1.2, as well as commercial products from Master Builders Solutions Deutschland GmbH via SEM (in gray): (e) X-Seed 100 compacted NPs used for SEM-EDX and (f) X-Seed 500 compacted NPs used for SEM-EDX; (g) X-Seed 100 single NPs; (h) X-Seed 500 single NPs.

The four C-S-H had contrasting morphology that obeyed a systematic evolution with the nominal Ca/Si ratio (Figure 1a–d). The sample with the lowest Ca/Si ratio, i.e., C-S-H 0.6, was composed of two different types of crystals that could be distinguished based on their morphology. Crystals of type 1 were seldom observed and could be described as nanosized crystals forming spheres with sizes on the order of 200–500 nm. The size of the individual crystals building up these spheres could hardly be determined because of the high degree of compaction but probably ranged between 10 and 50 nm. Crystals of type 2, which were by far the most abundant, could be described as crystals generally having sizes of 100–200 nm in the plane layer and 5–10 nm perpendicularly. The habit is close to automorphic, with the frequent presence of well-defined angles and edges. Both types of crystals had a SAED pattern typical for C-S-H, with the main diffraction maximum occurring at ∼3.1 Å. C-S-H 0.8 had close morphological similarities with C-S-H 0.6, albeit with type 1 crystals being even rarer, and type 2 crystals having rounder edges and less well-defined borders. In C-S-H 1.0, crystals of type 1 were not observed. Crystals of type 2 were still present but were approximately equally abundant to a new type 3 crystal, which could be described as having “rumpled sheet of paper” morphology. The size of these crystals could not be determined, due to the high degree of distortion, but was probably on the order of 200 nm in the layer plane and 10 nm perpendicularly. In C-S-H 1.2, type 3 crystals were the most abundant and accompanied by rare occurrences of type 2 crystals, which however had a habit that tended to change from automorphic to xenomorphic.

#### SEM Observations: X-Seed Morphology

The two X-Seed samples showed contrasting morphologies (Figure 1e–h). The dry powder of X-Seed 100 showed plate/sheet-shaped particle structures merged to a block of agglomerates in the nano- to micrometer range (Figure 1e). Single particles of X-Seed 100 were mainly spherical NPs (Figure 1g). In contrast, the dry powder of X-Seed 500 was composed of compacted NPs of two different morphologies (Figure 1f). The first morphology was needle-like, and the second one showed nearly spherical NPs in the nanometer range, crumpled up to the micrometer range. Single NPs of X-Seed 500 confirmed the versatile morphologies from spherical and square to undefined shapes in the nanometer range (Figure 1h). Ca/Si ratios via EDX were on average 2.1 for X-Seed 100 and 2.3 for X-Seed 500 (one example of each is shown in Figures S2 and S3).

#### Elemental Composition: C-S-H and X-Seed 100/500

**Figure 2:**
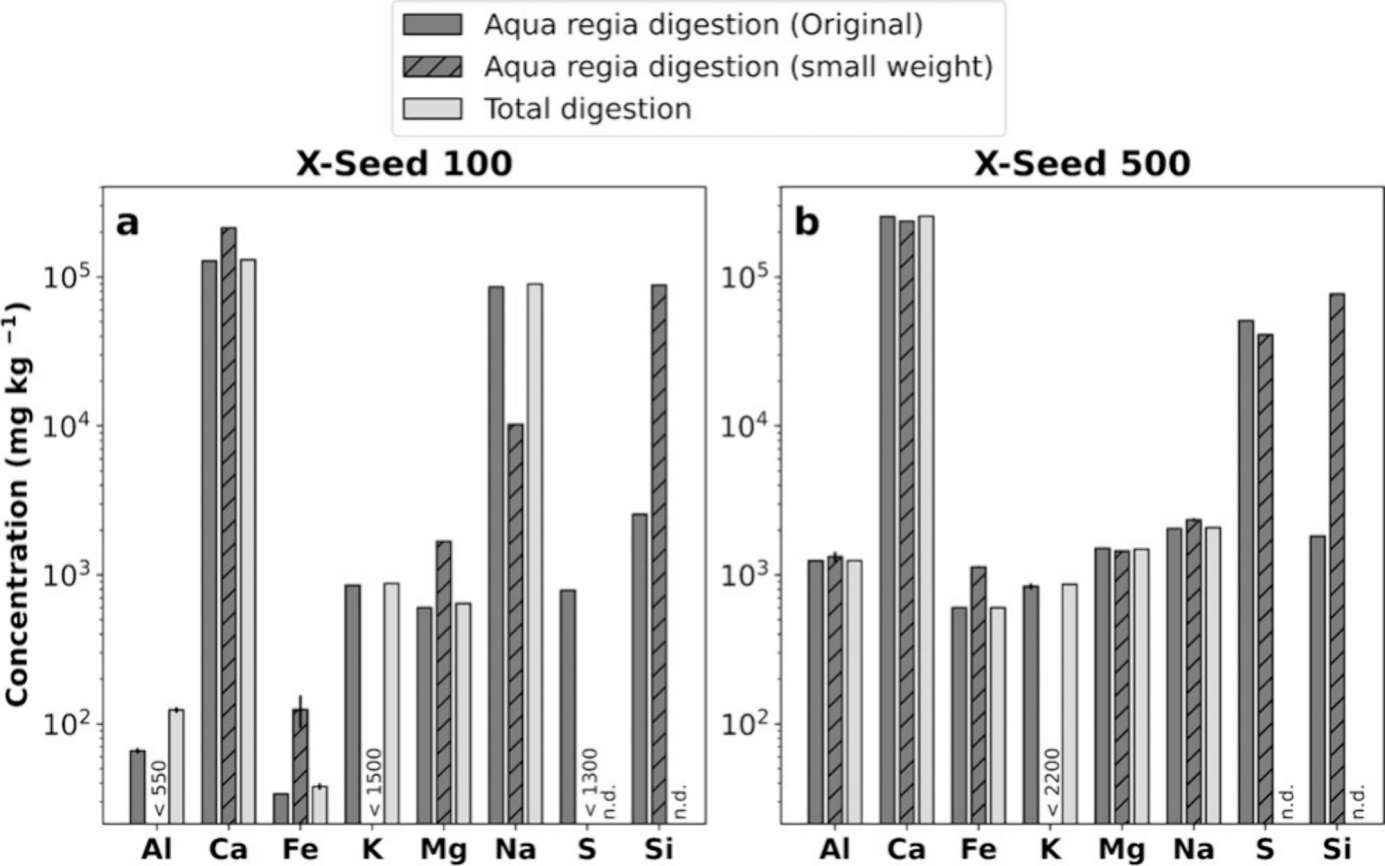
Chemical characterization via acid digestions (aqua regia and HF) was analyzed using bulk ICP-OES. The small weight of X-Seed 100 was ethanol-washed to remove the pore solution. Numbers represent the cases where results were below the limit of quantification (LOQ). n.d. = not determined. (a) X-Seed 100 and (b) X-Seed 500.

The C-S-H are hydration products of the reaction of CaO and SiO_2_ precursors, mixed in an aqueous solution in a CO_2_-free glovebox (Table S3). In the range of Ca/Si investigated here, the C-S-H Ca/Si is expected to follow closely the proportion of Ca and Si used for the reactants. As the elemental compositions of the X-Seed samples are unknown, we did a bulk ICP-OES measurement of the aqua regia and total digestions (Figure 2). Results show a diverse composition of the X-Seed material, i.e., not only pure Ca and Si compounds. For example, a much higher Na concentration was observed for X-Seed 100 than 500 and confirmed by SEM-EDX (Figure S4). NaNO_3_ is an artifact, precipitating when drying X-Seed 100, necessary for SEM-EDX. The producer specifies that supplements of NaNO_3_ (ca. 1%) are part of the X-Seed 100 sample production to reduce stress corrosion and setting time of concrete.^36^ Al, Fe, and S are lower in the X-Seed 100 sample than in the X-Seed 500 sample. The greater S in the X-Seed 500 is due to the fact that CaSO_4_ was added to the product instead of NaNO_3_. This suits the slightly greater Ca concentration in the X-Seed 500. Overall, both total and aqua regia digestions were in good agreement. Please note that Si and S were not determined after total digestion because of potential loss during the evaporation step in the form of volatile fluorides (Si as SiF_4(g)_^37^ and S as SF_6(g)_^38^). As Si is poorly soluble in aqua regia but cannot be quantified after total digestion, we additionally digested only 2–3 mg of the ethanol-washed X-Seed 100 NPs (to remove the pore solution) and of the X-Seed 500 dry powder (Figure 2 and Table S7). Since significantly higher Si concentrations were obtained (complete digestion) for the low-weight samples, those results were used to calculate the Ca/Si ratios.

### Method Development/Validation for spICP-MS

In the following sections, we describe the most important steps and results regarding method development and validation for the spICP-MS method in ethanol matrix. As the mass per particle is calculated from the raw particle signal(s) and the sizes emerge from the masses assuming spherical particle shapes, we use the particle sizes as a final step of method validation. PNC and Ca/Si ratios obtained by spICP-MS are compared with complementary methods for method validation, as they are important parameters for cement characterization.

#### Transport Efficiency

**Figure 3:**
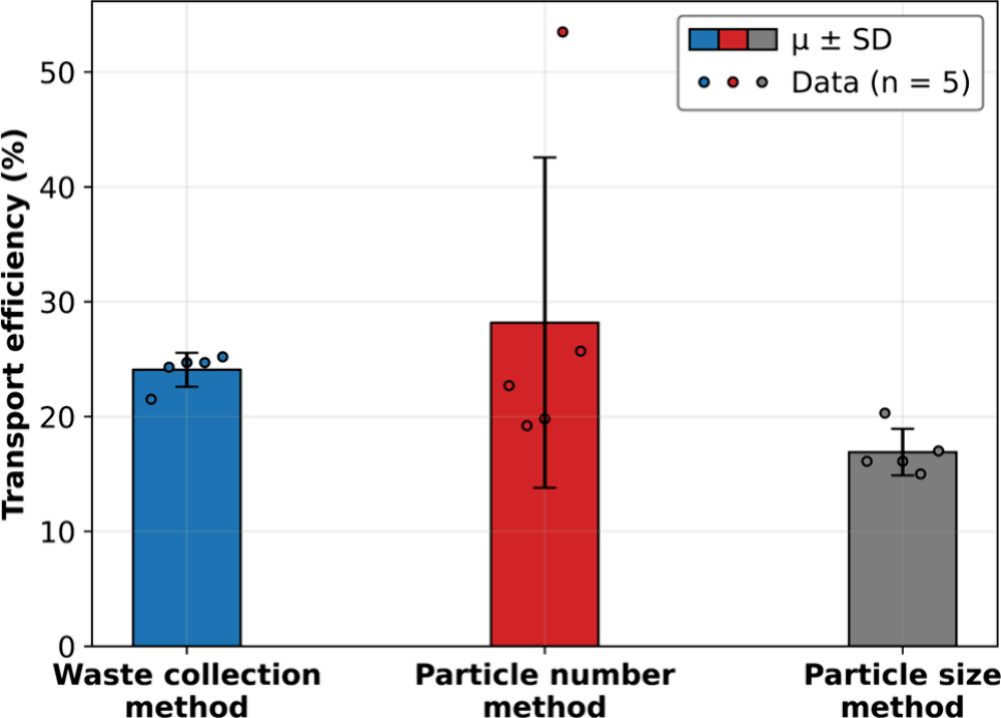
Transport efficiency comparison using three methods. The bars correspond to the average (μ) ± standard deviation (SD) (*k* = 1), and the circles correspond to each single data point (*n* = 5).

The TE is a crucial parameter, especially for polydispersed particle samples. The higher the TE, the more NPs from the suspension reach the plasma and therefore the lower the risk of missing NPs. TE was determined and compared using three methods: particle number, particle size, and waste collection method as described elsewhere^30^ (Figure 3). Even if, in previous studies, the waste collection method was not recommended because of uncertainties resulting from solution evaporation, our evaporation-sealed setup allowed us to accurately determine TE via sample and waste flow.^30^ Thus, for all calculations, TE was determined using the waste collection method and varied between 21.5 and 25.2%, which is much higher for this type of spray chamber than usually found for water matrix (2–10%).^9^

#### spICP-MS Validation: Linearities, LOD/LOQ, and Background

Calibration curves showed good linearity in pure ethanol with coefficients of determination (*R*^2^) ≥ 0.99 and for interference-rich elements ^28^Si^+^, ^40^Ca^+^, and ^32→48^S^+^ ≥ 0.93 (one example including ionic calibration parameters, ionic LODs, and LOQs is shown in Table S8). Especially for Ca and Si, the background is systematically higher (e.g., ^40^Ca^+^ interferes with ^40^Ar^+^). To prove this point, an overview of ethanol measured as blank and their spurious NPs as their corresponding sizes and PNC can be found in Figure S5.

### Particle Size Distribution Comparison: spICP-MS vs NTA, TEM, and SEM

#### Gold (Au)

**Figure 4:**
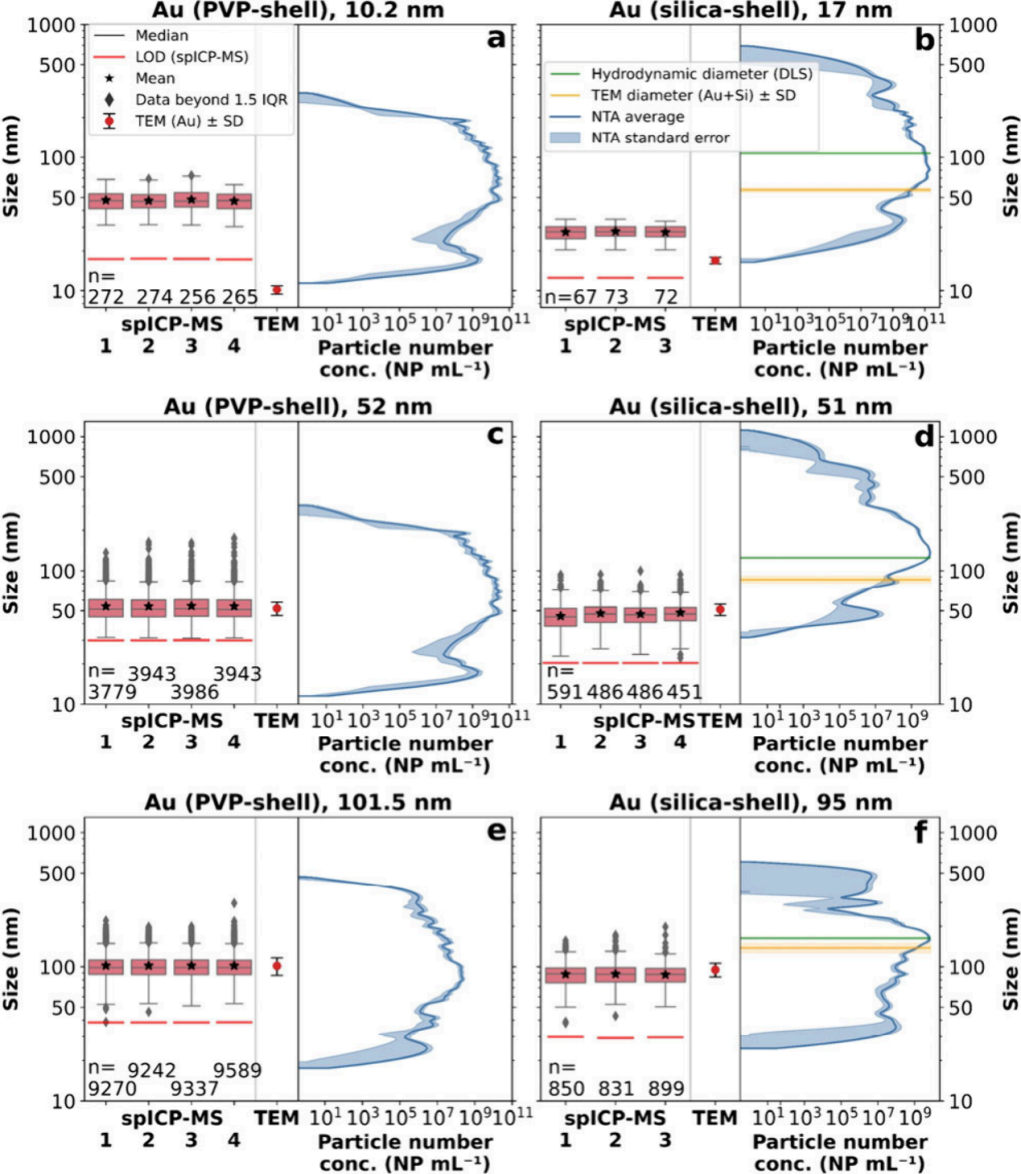
Size comparison (logarithmic) for the Au reference materials between spICP-MS, NTA, and TEM (certificate, nanoComposix). The numbers (1–3, 4) on the horizontal axis correspond to the replicates of spICP-MS. TEM error corresponds to the standard deviation (*k* = 1) and NTA error to the standard error (*k* = 1). The green line represents the hydrodynamic diameter analyzed by dynamic light scattering (DLS) and the yellow line is the TEM diameter (Au core + Si shell (both from the certificate, nanoComposix and independent from the PNC (horizontal axis)). (a) 10.2 nm (PVP-shell). (b) 17 nm (Au-core) and 57 nm total diameter (including silica-shell). (c) 52 nm (PVP-shell). (d) 51 nm (Au-core) and 86 nm total diameter (including silica-shell). (e) 101.5 nm (PVP-shell). (f) 95 nm (Au-core) and 138 nm (including silica-shell).

Common NPs to validate a spICP-MS method are Au NPs because of the high sensitivity of Au determination and the low number of potential interferences. Au can be considered a low background element due to its low solubility with a blank signal less than 50,000 cps (usually <2000 cps). Thus, the Poisson approach was used to determine the particle detection threshold (PDT) and to separate particle signals from the (ionic) background. However, for the small NPs (10 and 17 nm), the Gaussian approach was used, as it had a better-fitting particle detection threshold. The measured size distributions for Au NPs ≥ 51 nm via spICP-MS are in good agreement with the certified TEM sizes from nanoComposix (Figure 4c–f).

However, the small NPs (10 and 17 nm) were overestimated via spICP-MS, showing medians of ca. 47 nm (Au-PVP 10.2 nm, Figure 4a) and ca. 28 nm (Au–Si 17 nm, Figure 4b). Small NPs tend to be undistinguishable from the background/noise, in agreement with previous literature defining the size detection limit for Au as 20 nm.^39^ Also, those sizes were close to or below our size LOD and therefore could not be validated. In addition, smaller NPs likely showed a higher agglomeration potential than bigger NPs, despite bath sonication, especially clear in the NTA distributions (Figure 4a,b). Nevertheless, spICP-MS showed very good precision for the replicates measured for all materials. NTA size distributions were generally larger than spICP-MS but showed a similar trend, also overestimating small NPs < 51 nm: median of ca. 71.7 nm (Au-PVP 10.2 nm). NTA results obtained on the Au silica-shelled NPs (Figure 4b,d,f) were not directly comparable to spICP-MS, as NTA cannot distinguish the Au core from the silica shell. Still, Au silica-shelled NPs were measured to show the feasibility of using NTA as a comparison and validation method. Except for the 10.2 nm NPs, NTA was in very good agreement for all NPs with the total diameter (TEM) but also with hydrodynamic diameter (DLS). Moreover, PVP-shelled Au NPs were delivered and certified as powder and are certified in dry form via TEM.

#### Silica (SiO_2_) and Magnetite (Fe_3_O_4_)

**Figure 5:**
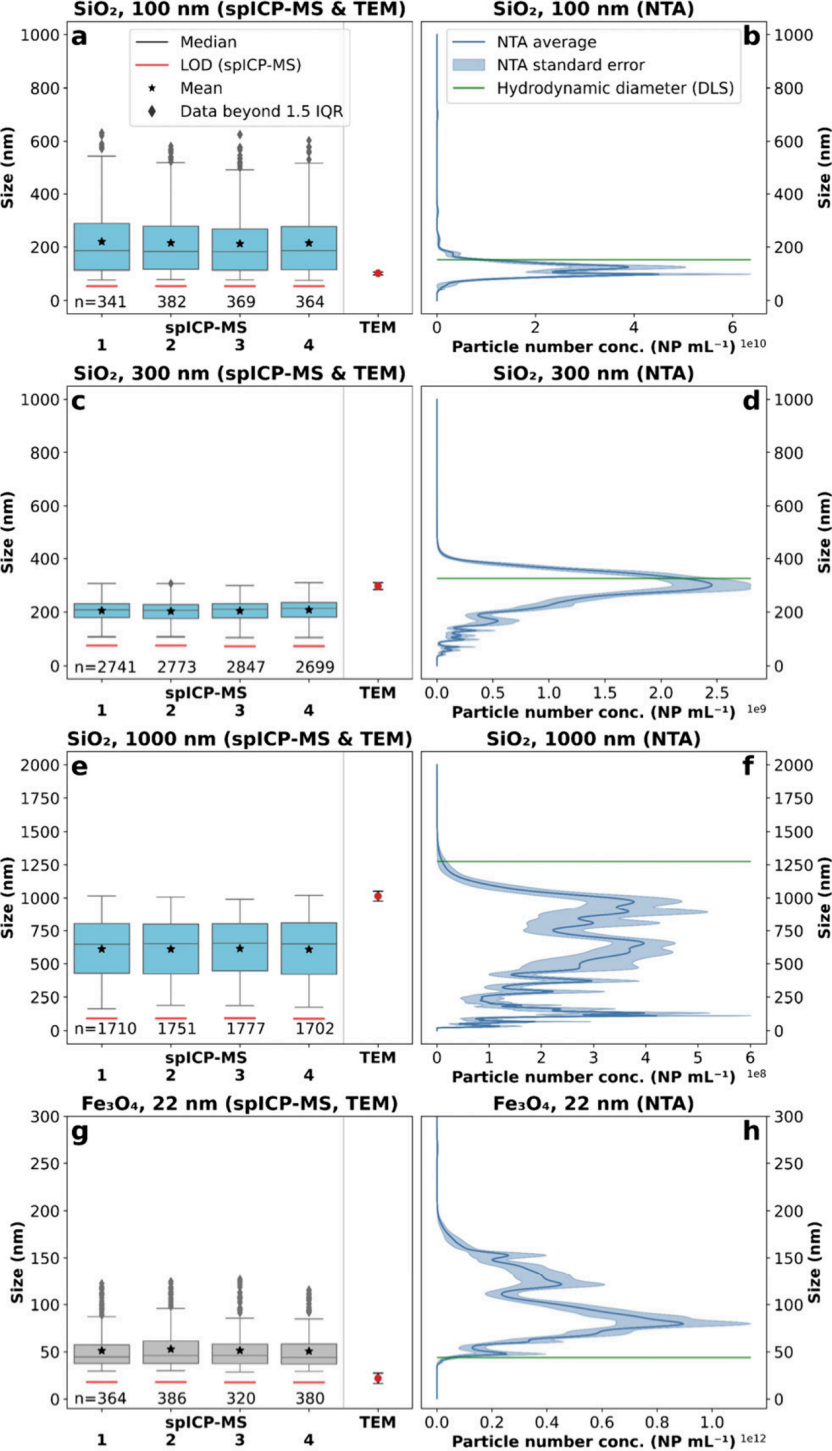
Size comparison for the SiO_2_ and Fe_3_O_4_ reference materials between spICP-MS, NTA, and TEM (certificate, nanoComposix). The numbers (1–4) on the horizontal axis correspond to the replicates of spICP-MS. TEM error corresponds to the standard deviation (*k* = 1) and NTA error to the standard error (*k* = 1). The green line corresponds to the hydrodynamic diameter (certificate, nanoComposix) and was measured via DLS. (a) 100 nm (aminated) via spICP-MS and TEM. (b) 100 nm (aminated) via NTA. (c) 300 nm (aminated) via spICP-MS and TEM. (c) 300 nm (aminated) via NTA. (e) 1000 nm (aminated) via spICP-MS and TEM. (f) 1000 nm (aminated) via NTA. (g) 22 nm (PVP-shell) via spICP-MS and TEM. (h) 22 nm (PVP-shell) via NTA.

The SiO_2_ and Fe_3_O_4_ NP sizes obtained from spICP-MS were generally in good agreement with the comparison methods via NTA and TEM (Figure 5a–h), though less precise than for Au.

The main challenge for the Si measurements was the high Si background in the samples due to glass containing ICP-MS equipment and ICP-MS-based interferences at *m*/*z* 28 and because samples were prepared in glass volumetric flasks to improve deagglomeration during bath sonication. These factors increased the LOD by ten times (SiO_2_: 200 nm) compared to Au analyses.^39^ Thus, the 100 nm SiO_2_ NPs could not be distinguished from the background using spICP-MS (median: ca. 185 nm; Figure 5a). A similar size distribution for the samples as in the blanks was observed (median: ca. 200 nm; Figure S5a), suggesting that the detected NPs are strongly biased by background spurious NPs. In this case, the NTA would be the preferable method to determine SiO_2_ NP sizes, as they showed good agreement (median: 116.5 nm) with the certified TEM diameter (Figure 5a,b). A previous publication reported a size LOD for NTA of 50 nm, valid for materials with comparably high refractive indices (RI) such as SiO_2_ (RI(SiO_2_) = 1.50).^40^ This outcome also applies to the 300 nm SiO_2_ NPs, where spICP-MS underestimates the particle sizes (median: ca. 225 nm) as the background NPs are undistinguishable from the aimed NPs. NTA was again in good agreement with the certified TEM diameter (median: 292.5 nm). However, when evaluating the 1000 nm SiO_2_ NPs, spICP-MS is in good agreement with NTA (spICP-MS median of ca. 650 nm vs NTA median of 650.8 nm), with, especially for the NTA, a wide particle distribution from 10 to 2000 nm. Only in that case, both methods highly underestimated the certified 1000 nm TEM diameter. For spICP-MS, this can again be explained by the poor discrimination and bias toward smaller NPs originating from the background. Another potential explanation for the size underestimation of big NPs is sedimentation. This does not seem to play a role in spICP-MS, as the fourth replicate (measured last) shows a comparable size distribution with the first replicate. For NTA, the overall measurement time (including optimization and preparation) is about seven times higher than spICP-MS (NTA: ≥15 min; spICP-MS: ca. 2 min), and settling of the biggest NPs might bias the NTA results. Nevertheless, a more likely explanation is that light scattering artifacts lead to spurious NPs and thus result in an underestimation of the particle sizes which is especially observed when analyzing comparably large particles.^41^ Generally, the sizes measured by NTA are closer to the certified TEM diameters than the certified hydrodynamic diameters measured by dynamic light scattering (DLS) (Table S2). This is because DLS overestimates especially polydisperse samples biasing larger NPs, as it is a bulk scattering intensity method, while NTA analyses single NPs and is a better method for polydisperse samples than DLS.^42^

Regarding the Fe_3_O_4_ NP analyses, both spICP-MS (median: ca. 45 nm) and especially the NTA (median: 97.4 nm) approach showed bigger sizes than the certified values of 22 nm by TEM (Figure 5g,h). This outcome could be related to the size LOD of the technique. A realistic (not calculated) size LOD reported in previous literature ranged between 40 and 50 nm for Fe_3_O_4_, close to the spICP-MS median value measured in this work.^23^ Additionally, Fe_3_O_4_ NPs are magnetic and tend to agglomerate in suspension, even in ethanolic matrices. As the measurement time of NTA is longer than that of spICP-MS, there is greater potential for agglomeration, which may be why spICP-MS sizes are smaller and closer to the certified TEM than results from the NTA. For instance, during the spICP-MS measurement, no further agglomeration could be detected, as replicate 1 showed a comparable size distribution to replicate 4. Generally, for all SiO_2_ and Fe_3_O_4_ NPs, the precision of spICP-MS replicates was also very good, as already presented for the case of Au NPs.

#### C-S-H Phases

**Figure 6:**
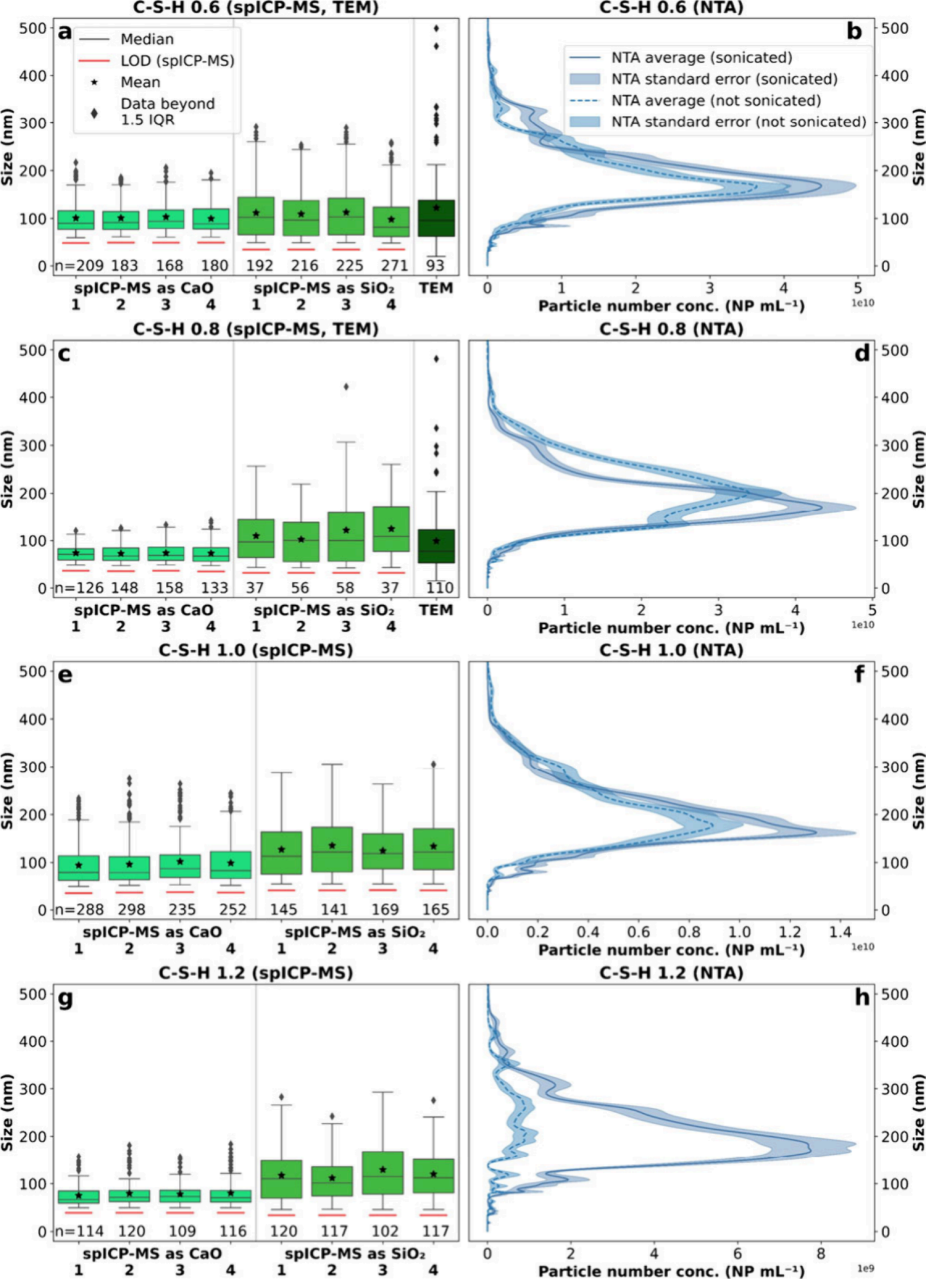
Comparison of C-S-H sizes obtained by spICP-MS, TEM (our data), and NTA. The numbers (1–4) on the horizontal axis correspond to the replicates of spICP-MS. NTA error corresponds to the standard error (*k* = 1). NTA was compared after ≥15 min sonication (20 °C) and without sonication. (a) C-S-H 0.6 via spICP-MS and TEM. (b) C-S-H 0.6 via NTA. (c) C-S-H 0.8 via spICP-MS and TEM; no TEM sizes could be measured as the NPs were too agglomerated and corrugated. (d) C-S-H 0.8 via NTA. (e) C-S-H 1.0 via spICP-MS and TEM; no TEM sizes could be measured as the NPs were too agglomerated and corrugated. (f) C-S-H 1.0 via NTA. (g) C-S-H 1.2 via spICP-MS and TEM; no TEM sizes could be measured as the NPs were too agglomerated and corrugated. (h) C-S-H 1.2 via NTA.

The four C-S-H were investigated via spICP-MS by analyzing Ca, expressed as CaO, and Si, expressed as SiO_2_ equivalent sizes. As these samples were not certified, particle sizes were validated with NTA and TEM analyses (Figure 6a–h).

However, TEM sizes could only be determined for C-S-H 0.6 and 0.8 (Figure 6a,c), as C-S-H 1.0 and 1.2 were already too agglomerated (see TEM images, Figure 1c,d). The agglomeration can be explained by the increasing Ca concentration with increasing Ca/Si (Table S3). Based on the Derjaguin, Landau, Verwey, and Overbeek (DLVO) theory, divalent cations such as “bridging” Ca^2+^ tend to agglomerate NPs due to attractive electrostatic forces.^43^ Another possible explanation is the fact that C-S-H 0.6 and 0.8 have a Ca/Si close to the ideal, defect-free, tobermorite-like structure and hence probably low permanent layer charge. This is not the case at higher Ca/Si, where the structure contains Si vacancies and hence a permanent layer charge that may favor agglomeration. For C-S-H 0.6 and 0.8, TEM and spICP-MS are in good agreement, thus we could validate spICP-MS also for synthetic cement samples. This result can be implicitly transferred for C-S-H 1.0 and 1.2, assuming that our spICP-MS also represents the correct sizes. In any case, all samples showed generally larger NPs based on Si as SiO_2_ than Ca as CaO, which might be a result of larger background NPs as discussed in the previous section for SiO_2_ NPs (Figure S5).

Regarding the NTA approaches, we showed in the previous sections that NTA was in good agreement with the CRM sizes for an ethanol matrix. Therefore, we can use the NTA results for the unknown samples with a similar matrix (e.g., C-S-H) as a reference method for size. Overall, the NTA size distributions were generally slightly larger than TEM and spICP-MS. This is thought to be because of the automorphic morphology with well-defined angles and edges which would lead to particle rotation during NTA analysis. As a consequence, the flow resistance is increased, leading to lower diffusion coefficients and thus resulting in an overestimation of particle sizes.

Nevertheless, the NTA measurements were also used to evaluate the bath sonication effect (Figure 6b,d,f,h). Under such conditions, we found that C-S-H 0.6, 0.8, and 1.0 showed similar size distributions and PNCs before and after ≥15 min bath sonication at 20 °C. However, for the most agglomerated sample C-S-H 1.2, a trend toward lower sizes and higher PNC after sonication was observed (median of 237 nm for not sonicated vs. 195 nm for sonicated samples; Figure 6h), showing the relevance of sonication with critical particle size.

#### X-Seed 100 and 500

**Figure 7:**
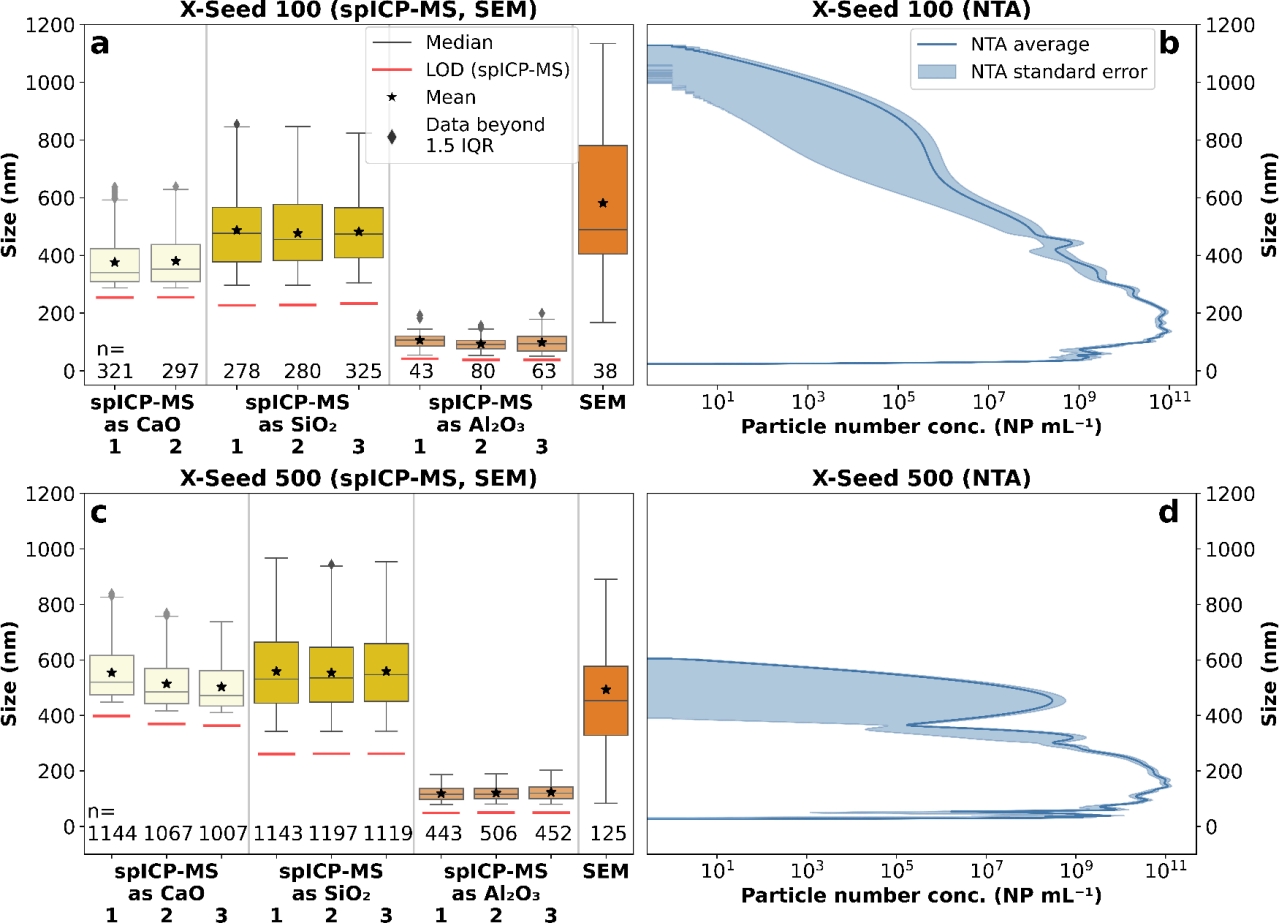
Size comparison for the industrial cement hardening accelerator products between spICP-MS, SEM and NTA. The numbers (1–3) on the horizontal axis correspond to the replicates of spICP-MS. NTA error corresponds to the standard error (*k* = 1). (a) X-Seed 100 via spICP-MS. (b) X-Seed 100 via NTA (semilogarithmic). (c) X-Seed 500 via spICP-MS. (d) X-Seed 500 via NTA (semilogarithmic).

The two industrial cement hardening accelerators showed versatile size distributions when calculating sizes based on the differentiated element of interest, which is possible via spICP-MS (Figure 7a,c).

We focused our spICP-MS results on the most important elements for cement: Ca, Si, and Al. As for the C-S-H, Ca- and Si-based X-Seed NPs are expected to be present as C-S-H phases. Therefore, we calculated their sizes as CaO and SiO_2_ equivalents. Al is often added as nano-Al_2_O_3_ (20–50 nm) to increase the compressive strength of concrete.^44^ Thus, we present Al as Al_2_O_3_ equivalent sizes too. X-Seed 100 spICP-MS size distributions were in good agreement with the SEM distributions (median: ca. 490 nm) for CaO (median: ca. 345 nm) and SiO_2_ (median: ca. 470 nm), while Al_2_O_3_ (median ca. 100 nm) sizes were significantly lower (Figure 7a). These Al-containing NPs could be either calcium aluminates or unreacted Al_2_O_3_, both detected as separate NPs. They could also be calcium aluminosilicate hydrates (C-A-S-H), where the Al is part of the same Ca- and Si-containing NPs but in much lower proportions, resulting in lower Al_2_O_3_ equivalent sizes. All spICP-MS and SEM sizes were within the NTA-range (23–1130 nm) even if NTA sizes were smaller in both samples (Figure 7b). For X-Seed 500, spICP-MS size distributions were in good agreement with SEM (median: 454 nm) for CaO (median: ca. 490 nm) and SiO_2_ (median: ca. 540 nm), while Al_2_O_3_ (ca. 120 nm) size distributions were again significantly lower (Figure 7c). Our SEM-EDX results potentially indicate the presence of C-A-S-H in X-Seed 500, as traces of Al were found for the same NPs as Ca and Si (Figure S3). Even though, Al was not detected via SEM-EDX in X-Seed 100, this does not discard any C-A-S-H phases present in the sample. The Al content in X-Seed 100 may be below the LOD as the Al concentration was one order of magnitude lower for X-Seed 100 (ca. 100 mg kg^–1^) than in X-Seed 500 (ca. 1000 mg kg^–1^). In any case, NTA results for X Seed 500 (27–605 nm) showed a polydisperse and narrower size distribution compared to X-Seed 100, pointing out the elemental difference in NP composition detected only via spICP-MS (main peak at ca. 150 nm and second peak at ca. 450 nm; Figure 7d). In summary, even for unknown, industrial cement samples, spICP-MS could be successfully validated for quantifying NP sizes, highlighting size differences based on elemental compositions.

### Particle Number Concentration (PNC)

**Figure 8:**
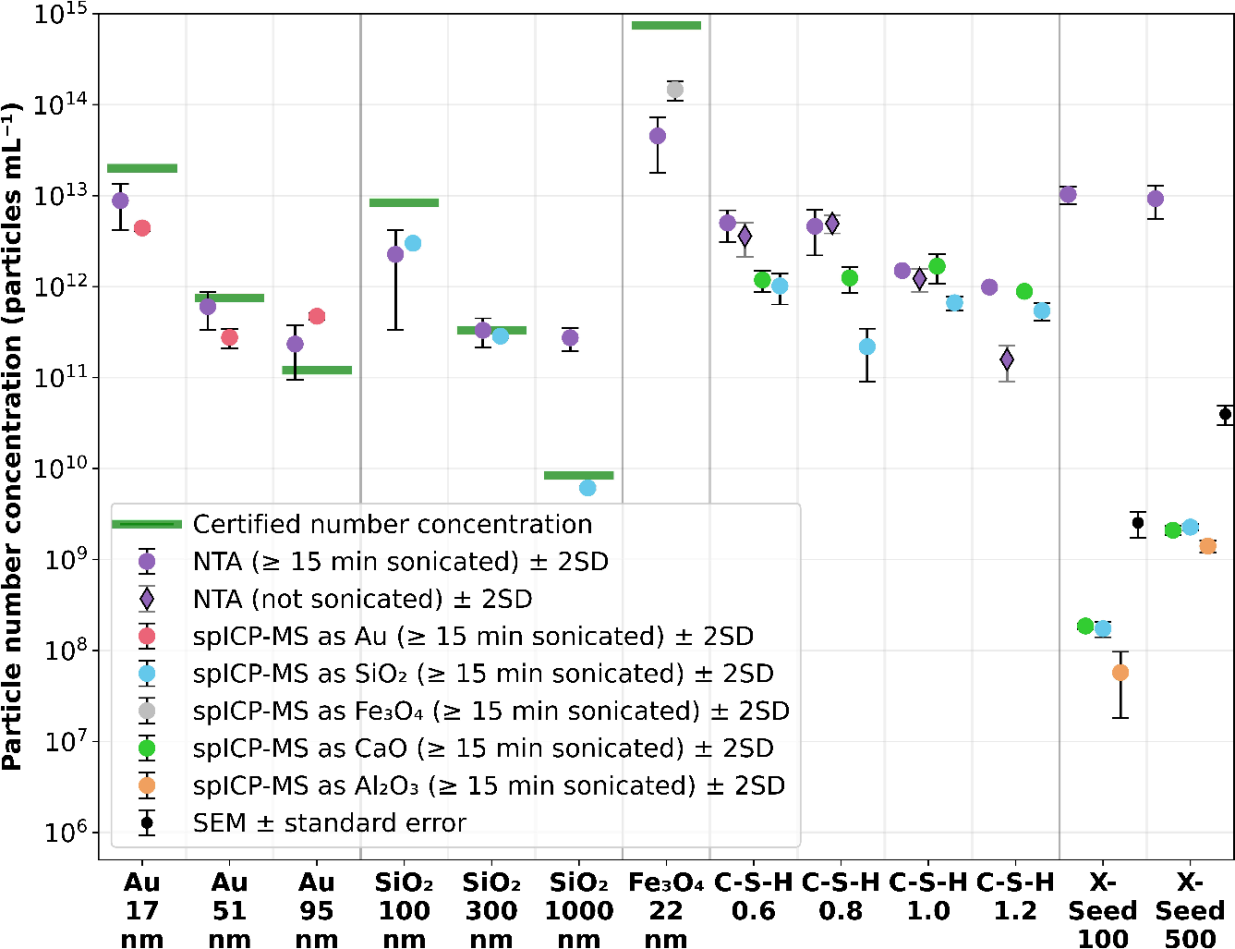
Particle number concentration comparison (logarithmic) between spICP-MS, NTA, SEM, and the certified values calculated via measured NP mass concentration (Gravimetric Analysis- AND HM-202), the average NP mass, and the volume per particle assuming a spherical shape. For spICP-MS, data represent the average ± 2SD of three to four replicates. For NTA, markers represent the average ± 2SD of the whole data set. For X-Seed 100 (spICP-MS as CaO) only two replicates were available; therefore the error bar represents the range instead of 2SD. For X-Seed 500 SEM, the error bar represents the range of two averages with different dilution factors (total number of measurements = 19).

Furthermore, we aimed to estimate the correct order of magnitude of PNC via spICP-MS (Figure 8).

While for the Au NPs, NTA and spICP-MS results were in the same order of magnitude, with NTA slightly closer to the certified number concentrations, for SiO_2_ and Fe_3_O_4_ NPs, spICP-MS was more precise and closer to the certified values. Especially for SiO_2_ (1000 nm), NTA overestimated the number concentration, probably because of the comparably large size and thus high amounts of light scattering leading to false particle detections. As for CRMs, spICP-MS and NTA were in good agreement with the certified values of PNC. Therefore, it can be assumed that also the unknown C-S-H and X-Seed samples have correct PNCs. For the C-S-H, we also checked the effect of bath sonication via NTA. When comparing our findings with the TEM images (Figure 1a–d), we only see a significant effect for the most agglomerated sample, C-S-H 1.2. In this case, bath sonication increased the PNC by almost one order of magnitude, indicating that temperature-controlled bath sonication does not have a negative effect but can have a positive effect on fast agglomerating samples. Finally, the industrial cement hardening accelerators (X-Seed 100 and 500) showed 4–5 orders of magnitude higher PNC for NTA than spICP-MS, while PNC estimated by SEM was around one order of magnitude higher than spICP-MS. To be sure that the majority of NPs were detected via SEM, the supernatant above the Si-wafer for SEM after centrifugation was analyzed using NTA. Some spurious NPs were detected but were within the SEM uncertainty. As both X-Seed samples consist of heterogeneous NPs containing different kinds of elements, NTA detects the whole spectrum of NPs, while spICP-MS can distinguish between elements and therefore only tracks NPs consisting of one chosen element (in this case, Si, Ca, and Al). However, it is also known that NTA can overestimate PNC, especially for very heterogeneous NPs (i.e., assumed to be the case for the X-Seed samples; Figure 7a–d) as not all NPs can be focused equally. The NTA detection limit might have been set too low, leading to the detection of “noise” and therefore to an overestimation of PNC.^45^ Overall, our results indicate that SEM is the method of choice for analyzing all heterogeneous NPs while NTA is well-suitable for homogeneous NPs in estimating the PNC. Nevertheless, spICP-MS allows us to differentiate between element-specific PNCs which opens up a completely new field of applications.

### Ca/Si Ratios

Being able to quantify Ca/Si ratios in C-S-H is important, as it controls the main mechanical (e.g., compressive strength) and chemical (e.g., equilibrium pH, [Ca], and [Si]) properties of these phases and that of cementitious materials.^46,47^ Here, C-S-H Ca/Si was studied with spICP-MS. For that, all NPs above PDT (separated from the background) were summed up and the molar concentrations were calculated (Table 3). The final Ca/Si ratios that emerged from those data were compared to the calculated (known) Ca/Si from the C-S-H. The calculated Ca/Si molar ratios from the synthesis of the C-S-H (Table S3) were verified using electron probe microanalysis (EPMA) in a previous publication.^28^

**Table 3:** Comparison of Calculated and spICP-MS C-S-H Ca/Si and of spICP-MS, XRF, SEM-EDX and Aqua Regia Digestion (ICP-OES) X-Seed Ca/Si^a^

	Molar Ca/Si ratios (NPs) (mol mol^–1^)				
sample	calculated	XRF	SEM-EDX^d^	aqua regia (ICP-OES)	spICP-MS
C-S-H 0.6	0.6	-	-	-	0.46
C-S-H 0.8	0.8	-	-	-	0.84
C-S-H 1.0	1.0	-	-	-	1.03
C-S-H 1.2	1.2	-	-	-	0.37
X-Seed 100	-	-	2.1	1.7	0.48
X-Seed 500	-	1.7,^b^ 1.5^c^	2.3	2.2,^b^ 1.7^c^	0.92

a Molar Ca/Si ratios (NPs) in mol mol^–1^.

b Total Ca/Si ratio (not corrected).

c X-Seed 500 showed around 51 g kg^–1^ sulfur. We have indices from the producer that sulfur is present as CaSO_4_. Taking this into account, we receive a Ca/Si ratio of 1.5 (XRF) and 1.7 (aqua regia, ICP-OES).

d SEM-EDX was applied to rough particle surfaces (not polished), which might lead to an increased error for those results.

spICP-MS Ca/Si ratios for the C-S-H samples were in good agreement with the nominal Ca/Si for C-S-H 0.6, 0.8, and 1.0. However, the spICP-MS Ca/Si ratio determined for C-S-H 1.2 was much lower than expected. The reason for that might be related to the high Si background during the spICP-MS measurement. If the sample was too diluted, the high Si (background) falsely detected NPs, overestimating the accumulated Si NP-concentration and therefore underestimating the Ca/Si ratio.

In contrast, the Ca/Si ratios for the X-Seed samples were unknown and therefore characterized by complementary methods such as XRF (Table S9), SEM-EDX, and aqua regia digestion. Ca/Si ratios for X-Seed 100 ranged between 1.7 and 2.1, whereas for X-Seed 500 it was between 1.7 and 2.3 These ratios are comparable to previous literature, where Ca/Si ratios for C-S-H in neat Portland cement paste varied between 1.2 and 2.3, with a mean of 1.75 analyzed as bulk.^48^ spICP-MS showed much lower Ca/Si ratios for both X-Seed samples (i.e., 0.48 for X-Seed 100 and 0.92 for X-Seed 500). In C-S-H, when Ca/Si increases from ∼0.6 to ∼1.2, the structure of the phases changes due to depolymerization of the Si wollastonite-like chains, creating a layer charge deficit resulting in a Ca-interlayer incorporation as Ca and Ca(OH)_2_.^49,50^ Even though Ca(OH)_2_ is part of C-S-H, it should be noted that bulk methods always receive a sum of C-S-H and crystalline Ca(OH)_2_ NPs, which result in increased Ca/Si ratios.^51^ spICP-MS results strongly suggest that we were able to separate the small Ca(OH)_2_ particles, e.g., due to bath sonication, from the C-S-H by releasing them into suspension so that we analyzed the C-S-H without the Ca(OH)_2_ interlayer. Such interpretation is supported by the fact that the reported X-Seed 100 equilibrium pH is 11, possibly due to the industrial organic and inorganic supplements, incompatible with a C-S-H Ca/Si > 1.7 that has an equilibrium pH of ∼12.5.^47^ Even when taking into account a pH uncertainty of one unit, as provided in the X-Seed 100 data sheet, the limits of possible Ca/Si range between ∼0.5 and 1.3. As discussed in the case of Ca(OH)_2_, small NPs are the limitations of spICP-MS and are removed within the background. Another explanation could be related to Ca dissolution in ethanol, resulting in an underestimation of Ca/Si, as all other complementary methods were done using solid NPs while spICP-MS was done in suspension. This is likely as the Ca background increased for the X-Seed samples compared to the C-S-H, also visible in Figure 7 from the high size LODs.

## Conclusions

Until recently, it was not possible to directly and element-specifically measure highly reactive or water-sensitive nanoparticles (NPs) and colloids, such as cementitious hydration product NPs, regarding their size, particle number concentration (PNC), and elemental composition. This study demonstrates for the first time a single particle (sp) ICP-MS method in pure ethanol capable of determining element-specific size distributions between ca. 50 and 1000 nm, the order of magnitude in PNC, and elemental ratios such as Ca/Si ratios of cementitious NPs. Compared to most other particle characterization methods according to size distributions and PNCs, spICP-MS is especially valuable for identifying elemental-specific size distributions within heterogeneous polydisperse NPs, as presented for the X-Seed samples. Furthermore, our method can also be applied as a quality control method for purity of NPs to check possible element-specific contaminations. We presented a method validated for seven elements (Mg, Al, Si, S, Ca, Fe, and Au) with a potential of easily being extended to other elements of interest measurable by ICP-MS. Despite limitations to our spICP-MS method for small NPs ≤ 50 nm, especially for high-background elements such as Si or Ca, we believe that our method contributes to a valuable extent to the current state-of-the-art in characterizing all sorts of fast nucleation (nano)particles, providing a robust and wide spectrum of results without elaborate sample preparation. Moreover, ethanol not only stops the hydration reaction of cementitious NPs, but combined with bath sonication, it is also a strong dispersant agent to reduce NP agglomeration, despite the potential biases for specific cases such as particularly small Au, Fe_3_O_4_, and some C-S-H NPs. Such an approach broadens ongoing and future research fields related to the role of C-S-H phases in the sorption/transport of radionuclides from deep geological nuclear storage sites or the use of calcinated clays as an alternative for reducing the CO_2_ footprint of Ordinary Portland Cement.

## Supporting Information Available

Information about the standards and CRMs used in this work, the synthesis of C-S-H, the SEM PNC determination, the NTA Instruments settings, the elemental concentrations (X-Seeds) obtained via ICP-OES, as well as validation details (spICP-MS) and XRF results (X-Seed 500), ICP-MS/MS cell gas optimization parameters, SEM-EDX determination and spectra for X-Seeds, results on size and PNC of ethanol blanks (spICP-MS), equations to show calculations of PNC via SEM and of LOD_mass_ and LOD_size_ for the spICP-MS (PDF)
