# Supplementary material for: Characterization of Nanoparticles in Ethanolic Suspension Using Single Particle Inductively Coupled Plasma Mass Spectrometry: Application for Cementitious Systems
Source: ACS Omega. 2024 Jul 2;9(28):30294–307. doi: 10.1021/acsomega.4c01196 (PMC11256340; doi:10.1021/acsomega.4c01196)
Supplement: Supplementary file 1 — ao4c01196_si_001.pdf [file ao4c01196_si_001.pdf]

Characterization of nanoparticles in ethanolic suspension using spICP-MS: application for cementitious systems

Steffen Hellmann[a,b*], Teba Gil-Díaz[a†], Marcus Böhm[a], Dirk Merten[a], Sylvain Grangeon[c], Fabienne Warmont[d], Sophie Unbehau[e], Thomas Sowoidnich[e], Thorsten Schäfer[a*]

a Friedrich Schiller University Jena, Institute of Geosciences, Burgweg 11, 07749 Jena, Germany

b International Max Planck Research School for Global Biogeochemical Cycles, Max Planck Institute for Biogeochemistry, Department of Biogeochemical Processes, Hans-Knöll-Straße 10, 07745 Jena, Germany

c BRGM, 3 Avenue Claude-Guillemin, F-45060 Orléans, France

d ICMN, 1B, rue de la Férollerie CS40059, F-45071 Orléans Cedex 2, France

e Bauhaus-Universität Weimar, Institute for Building Materials, Coudraystr. 11, 99423 Weimar, Germany

† Present address: Karlsruhe Institute of Technology, Institute of Applied Geosciences, Adenauerring 20b, 76131, Karlsruhe, Germany

TABLES

Table S1: Ionic standards used for the element quantification via spICP-MS.

Standard description	Producer
Aluminium ICP standard 1,000 mg L^{-1} ($Al(NO_3)_3$, 2–3% HNO_3)	Certipur®, Merck, Darmstadt, Germany
Calcium ICP standard 1,000 mg L^{-1} ($Ca(NO_3)_2$, 2–3% HNO_3)	Certipur®, Merck, Darmstadt, Germany
Iron standard solution 1,000 mg L^{-1} ($Fe(NO_3)_3$, 0.5 mol L^{-1} HNO_3)	Certipur®, Merck, Darmstadt, Germany
Gold ICP standard 1,000 mg L^{-1} ($AuCl_4$, 7% HNO_3)	Certipur®, Merck, Darmstadt, Germany
Magnesium ICP standard 1,000 mg L^{-1} ($Mg(NO_3)_2$, 2–3% HNO_3)	Certipur®, Merck, Darmstadt, Germany
Silicon standard solution 1,000 mg L^{-1} ($(NH_4)_2SiF_6$, H_2O)	Certipur®, Merck, Darmstadt, Germany
Sulfur ICP standard 10,000 mg L^{-1} (from H_2SO_4, in H_2O)	Certipur®, Merck, Darmstadt, Germany

Table S2: Nanoparticle reference materials used for the method validation/calibration of spICP-MS.

Standard description	Element	Certified diameter (TEM) (nm)	Certified hydro-dynamic diameter (DLS in ethanol) (nm)	Matrix	Producer
10 nm NanoXact Gold Nanospheres – PVP (Dried)	Au	10.2 ± 0.8	-	Dry powder	nanoComposix, San Diego, USA
Silica Shelled 20 nm Gold Nanospheres, NanoXact™	Au	17 ± 1	107	Ethanol	nanoComposix, San Diego, USA
Silica Shelled 50 nm Gold Nanospheres, NanoXact™	Au	51 ± 5	125	Ethanol	nanoComposix, San Diego, USA
50 nm NanoXact Gold Nanospheres – PVP (Dried)	Au	52 ± 6	-	Dry powder	nanoComposix, San Diego, USA
Silica Shelled 100 nm Gold Nanospheres, NanoXact™	Au	95 ± 11	163	Ethanol	nanoComposix, San Diego, USA
100 nm NanoXact Gold Nanospheres – PVP (Dried)	Au	101.5 ± 15.1	-	Dry powder	nanoComposix, San Diego, USA
NanoXact 100 nm Silica Nanospheres – Aminated	Si	102.0 ± 5.3	152.5	Ethanol	nanoComposix, San Diego, USA
NanoXact 300 nm Silica Nanospheres – Aminated	Si	297 ± 13	326	Ethanol	nanoComposix, San Diego, USA
NanoXact 1 µm Silica Nanospheres – Aminated	Si	1014 ± 37	1276	Ethanol	nanoComposix, San Diego, USA
NanoXact 20 nm Magnetite Nanoparticles – PVP	Fe	22 ± 5.5	44	Aqueous 2 mM citrate	nanoComposix, San Diego, USA

Table S3. Synthesis of the calcium-silicate-hydrate (C-S-H) phases by BRGM (France). The final volume was 200 mL for calculating the particle concentrations.

Calculated Ca/Si molar ratio	CaO weight (g)	Ca		SiO_2 weight (g)	Si	
		Equilibrium concentration (mg L^{-1})	Particle concentration (mg L^{-1})		Equilibrium concentration (mg L^{-1})	Particle concentration (mg L^{-1})
0.6	1.45	$1.17 \cdot 10^{-3}$	$5.18 \cdot 10^{3}$	2.57	$2.87 \cdot 10^{-3}$	$6.01 \cdot 10^{3}$
0.8	1.71	$8.71 \cdot 10^{-4}$	$6.11 \cdot 10^{3}$	2.31	$9.33 \cdot 10^{-4}$	$5.40 \cdot 10^{3}$
1.0	1.93	$4.02 \cdot 10^{-3}$	$6.90 \cdot 10^{3}$	2.07	$3.41 \cdot 10^{-5}$	$4.84 \cdot 10^{3}$
1.2	2.12	$6.21 \cdot 10^{-3}$	$7.58 \cdot 10^{3}$	1.89	$2.23 \cdot 10^{-5}$	$4.42 \cdot 10^{3}$

Table S4: X-Seed 100 and 500 preparation for SEM size and particle number concentration (PNC) determination to estimate total dilution factors.

Sample description	Target DF	Empty weight (g)	Target volume (µL)	+Stock (g)	Stock volume (µL)*[1]	Total weight (g)	Total volume (mL)*[1]	DF	Total DF*[2]
X-Seed 100	10^5	14.4125	500	14.8287	527.30	53.6108	49.7	94	94181
	10^6	14.4208	50	14.4617	51.82	53.6143	49.7	958	958276
	10^7	14.3174	5	14.3234	7.60	53.7928	50.0	6579	6579233
X-Seed 500	10^5	14.4343	500	14.8455	520.97	53.5311	49.5	95	95080
	10^6	14.4254	50	14.4659	51.31	53.3111	49.3	960	960141
	10^7	14.3782	5	14.3816	4.31	53.4990	49.6	11506	11506118

*[1]: For the conversion from mass to volume a density of ethanol of 0.7893 g cm^{-3} (20 °C) was chosen.[1]

*[2]: The stock solution was already 1000 times diluted, thus the total dilution factor (DF) is 1000 times the DF.

Table S5: X-Seed 100 and 500 preparation for SEM size and particle number concentration (PNC) determination to estimate exact volumes.

Sample description	Target DF	Empty weight (g)	Target volume (mL)	Total weight (g)	Total volume (mL)*[1]
Blank (1)	1	41.4706	20	57.2773	20.0262
Blank (2)	1	41.2251	20	57.2184	20.2626
X-Seed 100	10^5	41.4547	20	57.5927	20.4460
X-Seed 100	10^6	41.4653	20	57.3450	20.1187
X-Seed 100	10^7	41.4402	20	57.2428	20.0210
X-Seed 500	10^5	41.4900	20	57.1933	19.8952
X-Seed 500	10^6	41.3863	20	57.1019	19.9108
X-Seed 500	10^7	41.4997	20	57.2875	20.0023

*[1]: For the conversion from mass to volume a density of ethanol of 0.7893 g cm^{-3} (20 °C) was chosen.[1]

Table S6: Chosen instrument settings for nanoparticle tracking analysis (NTA).

Parameter	Value
Temperature	20 °C
Dynamic viscosity (ethanol 20 °C)[2]	1.144 mPa s^{-1}
Laser type	Blue (488 nm)
Syringe pump speed	50–100*[1]
Min track length	10
Max jump distance	15
Detection threshold	5
Number of videos	20
Capture time	10 s

*[1]: Arbitrary unit according to the NTA operation software NanoSight 3.4.

Table S7: Elemental concentrations of X-Seed 100 (ethanol washed and dried at 40 °C) and 500 (dry powder) as mean ± standard deviation (SD) received via aqua regia digestion of 2–3 mg and measured via ICP-OES.

Element	X-Seed 100 (weight 0.0024 g)		X-Seed 500 (weight 0.0023 g)	
	Mean (mg kg^{-1})	SD (mg kg^{-1})	Mean (mg kg^{-1})	SD (mg kg^{-1})
Al	< 550	-	1326	109
Ca	213405.0	1042.0	237227	1957
Fe	125.0	31.0	1130	22
K	< 1500	-	< 2200	-
Mg	1684.0	6.0	1444	9
Mn	< 15	-	100	4
Na	10219.0	52.0	2348	65
S	< 1300	-	40935	109
Si	88125.0	417.0	76842	652
Sr	71.3	0.4	129	1

Table S8: Ionic limits of detection (LOD) and quantification (LOQ) comparison quartz (batch May 2022, Au (PVP-shell)) vs. borosilicate volumetric flasks (batch November 2021) for the spICP-MS method in ethanol.

Isotope	Volumetric flask glass type	LOD*1 ($\mu g\ L^{-1}$)	LOQ*1 ($\mu g\ L^{-1}$)	Slope (m) (CPS ($\mu g\ L^{-1})^{-1}$)	Intercept (b) (CPS)	Coefficient of determination (R^2)
$^{24}Mg^+ \rightarrow {}^{24}Mg^+$	Quartz	0.42	0.45	24857	0*2	0.9999
	Borosilicate	0.57	0.64	28494	0*2	0.9971
$^{27}Al^+ \rightarrow {}^{27}Al^+$	Quartz	0.18	0.22	27479	-2651	0.9999
	Borosilicate	0.51	0.63	39029	-14725	0.9994
$^{28}Si^+ \rightarrow {}^{28}Si^+$	Quartz	2.46	2.93	44596	111232	1.0000
	Borosilicate	14.5	29.9	9644	0*2	0.9999
$^{32}S^+ \rightarrow {}^{32}S^{16}O^+$	Quartz	9.47	10.4	624	7468	1.0000
	Borosilicate	4.13	5.83	610	10857	0.9998
$^{40}Ca^+ \rightarrow {}^{40}Ca^+$	Quartz	0.83	1.58	10936	-3509	0.9996
	Borosilicate	1.25	1.65	12335	0*2	1.0000
$^{56}Fe^+ \rightarrow {}^{56}Fe^+$	Quartz	0.61	0.77	53892	-11902	0.9996
	Borosilicate	1.44	1.48	72610	-95213	0.9973
$^{197}Au^+$	Quartz	0.185	0.185	29983	-5539	0.9999
	Borosilicate	0.307	0.312	37861	-11444	0.9995

*1: For the calculations, ≥ three blanks and the linear function ($y = m \cdot x+b$) of the calibration curve (of each element) were used to estimate the LODs/LOQs: LOD = 3·SD+mean; LOQ = 10·SD+mean).

*2: The calibration curve was forced through the origin if the intercept was higher than the blank signal.

Table S9: X-ray fluorescence (XRF) analysis for the industrial cement hardening accelerator X-Seed 500.

Mineral	Mass proportion (w%)	Molar mass (g mol^{-1})	Molar proportion (mol (mg sample)$^{-1}$)
SiO_2	24.51	60.08	4.08
TiO_2	0.02	79.87	< 0.01
Al_2O_3	0.27	101.96	0.03
Fe_2O_3	0.12	159.69	0.01
Cr_2O_3	< 0.01	151.99	< 0.01
MgO	0.25	40.30	0.06
CaO	39.53	56.08	7.05
SrO	0.01	103.62	< 0.01
K_2O	0.11	94.20	0.01
Na_2O	0.22	61.98	0.04
SO_3	7.14	80.06	0.89
P_2O_5	0.01	283.89	< 0.01
ZnO	< 0.01	81.38	< 0.01
MnO	0.04	70.94	0.01
Ignition loss	27.00	-	-
Sum	99.24	-	-

FIGURES

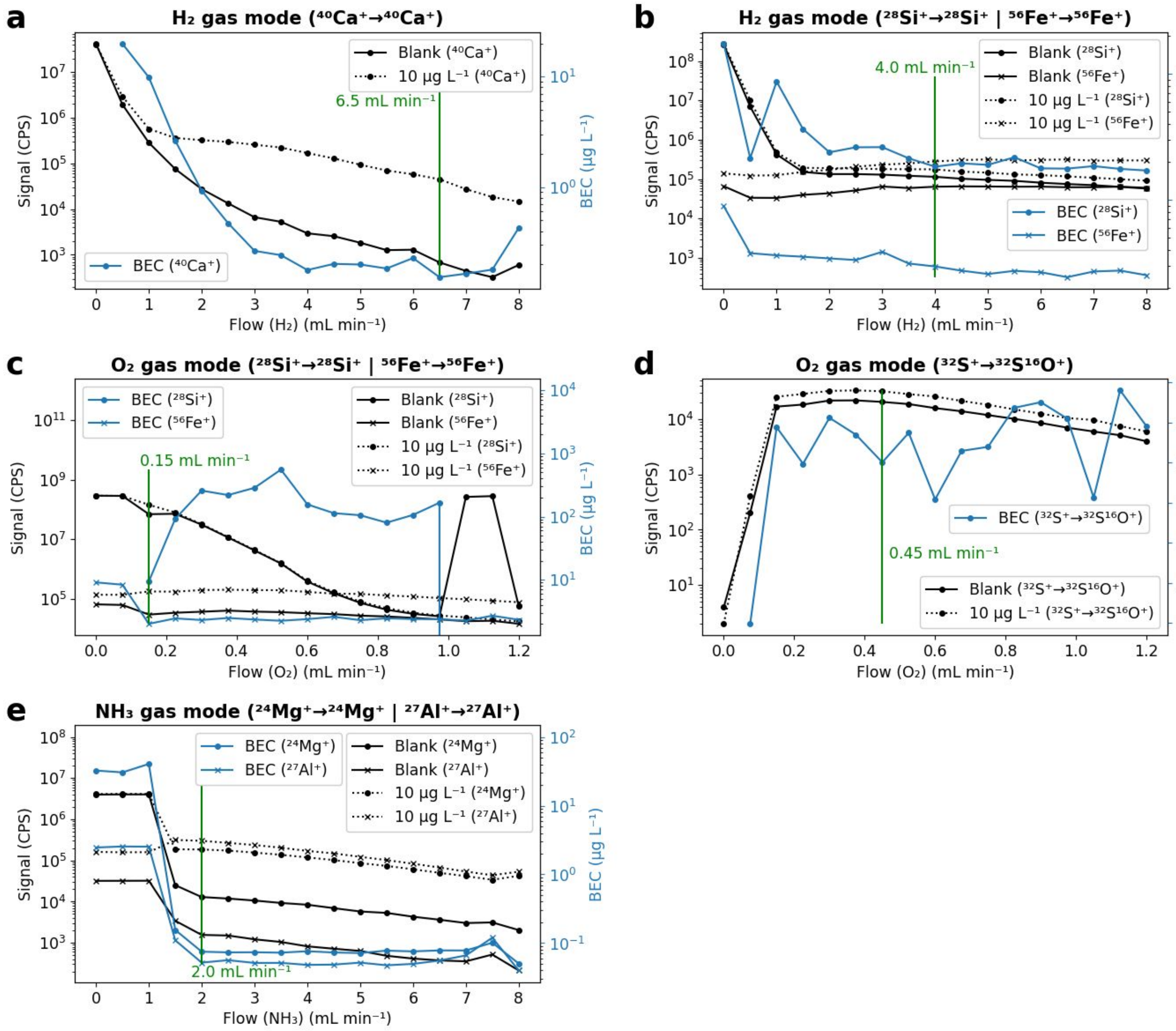

Figure S1. Cell gas flow optimisation of the ICP-MS/MS 8900 for single particle analysis. The green vertical lines correspond to the final optimised cell gas flow. (a) Gas flow optimisation for the H_2 cell gas mode used for $^{40}Ca^+$ analysis. (b) Gas flow optimisation for H_2 in the mixed $H_2 + O_2$ cell gas mode*¹ used for $^{28}Si^+$ and $^{56}Fe^+$ analysis. (c) Gas flow optimisation for O_2 in the mixed $H_2 + O_2$ cell gas mode*¹ used for $^{28}Si^+$ and $^{56}Fe^+$ analysis. (d) Gas flow optimisation for the O_2 cell gas mode used for $^{32}S^+ \rightarrow {}^{32}S^{16}O^+$ analysis. (e) Gas flow optimisation for NH_3 in the NH_3/He (90%/10%) cell gas mode used for $^{24}Mg^+$ and $^{27}Al^+$ analysis.*¹: In the cell gas optimisation mode it was only possible to optimise one gas after the other. Thus, in the mixed $H_2 + O_2$ mode, H_2 and O_2 were optimised in separate runs independently from each other.

(a) (b)

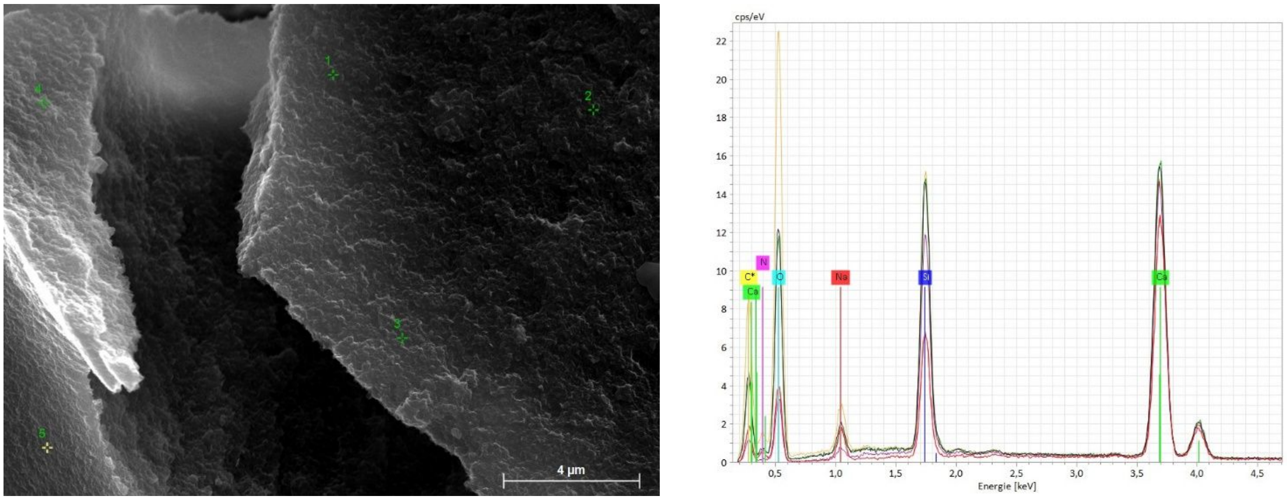

Figure S2. SEM-EDX of X-Seed 100 agglomerates to determine the molar Ca/Si ratio. (a) SEM image. (b) EDX spectrum.

(a) (b)

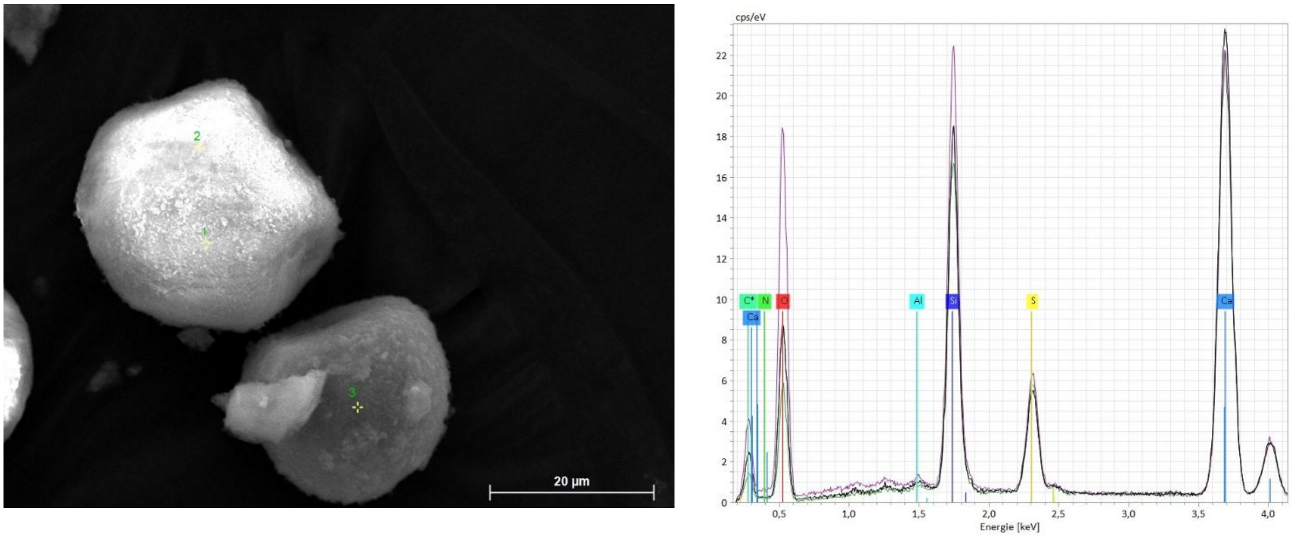

Figure S3. SEM-EDX of X-Seed 500 agglomerates to determine the molar Ca/Si ratio. (a) SEM image. (b) EDX spectrum.

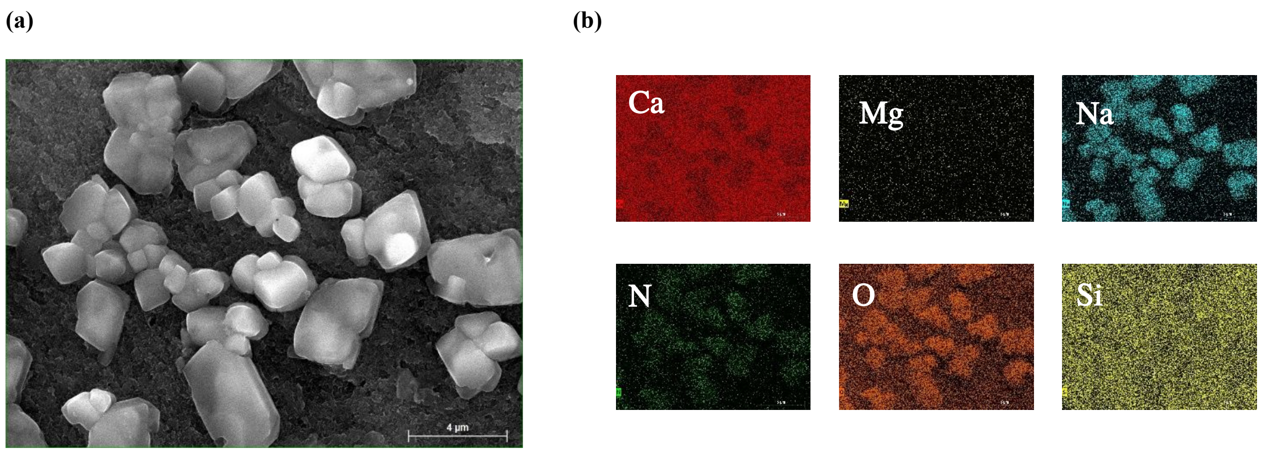

Figure S4. SEM-EDX mapping of X-Seed 100 precipitates to discover their composition (a) SEM image. (b) EDX mapping data for Ca, Mg, Na, N, O and Si.

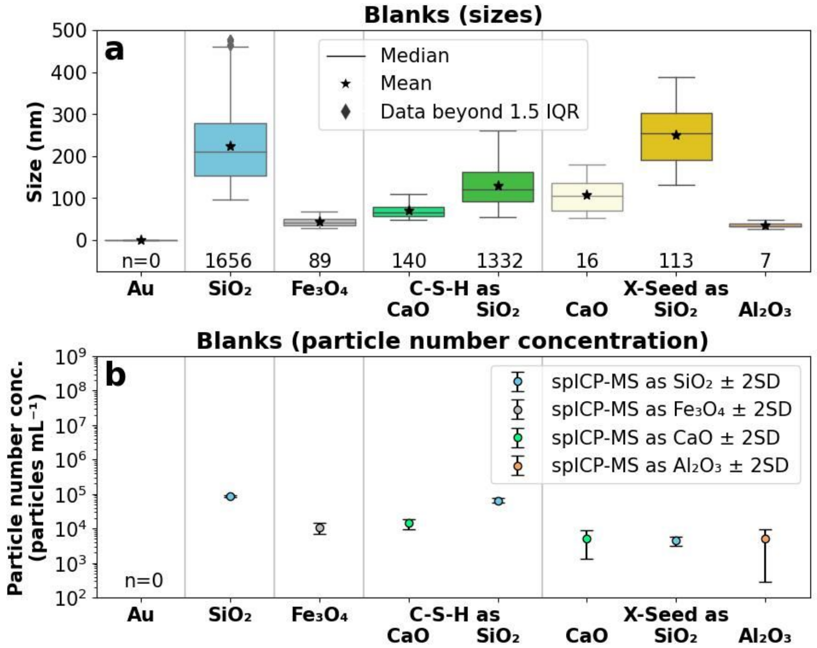

Figure S5. Ethanol was analysed as blank in each batch. "Au" represents ethanol measured on the same day as the Si-shelled Au NPs. "SiO_2" represents ethanol measured on the same day as the aminated SiO_2 reference material NPs. "Fe_3O_4" represents ethanol measured on the same day as the PVP-shelled Fe_3O_4 reference material NPs. "C-S-H as CaO/SiO_2" represents ethanol measured on the same day as the C-S-H phases. "X-Seed as $CaO/SiO_2/Al_2O_3$" represents ethanol measured on the same day as the commercial X-Seed samples. (a) Sizes. (b) Particle number concentration.

EQUATIONS

Equation S1. Calculation of particle number concentration via SEM (as an example for the two blanks):

$$h = \frac{V_{suspension}}{\pi \cdot (\frac{d}{2})^2} = \frac{20.1444\ cm^3}{\pi \cdot (\frac{2.46\ cm}{2})^2} = 4.24\ cm$$

$$V_{SEM\ image} = a \cdot b \cdot h = 257.7\ \mu m \cdot 381.7\ \mu m \cdot 4.24\ cm \cdot \frac{10000\ \mu m}{cm}$$

$$V_{SEM\ image} = 4170637416\ \mu m^3 \cdot 10^{-9}\ \frac{\mu L}{\mu m^3} = 4.171\ \mu L$$

$$PNC = \frac{N_{median}}{V_{SEM\ image}} \cdot DF = \frac{1}{4.171\ \mu L} \cdot \frac{1000\ \mu L}{mL} \cdot 1 = 240 \frac{NP}{mL}$$

Legend

$h = height\ of\ the\ volume\ above\ Si - waver\ [cm]$; $d = inner\ diameter\ of\ the\ centrifugation\ tube\ [cm]$; $V_{suspension} = volume\ of\ suspension\ [cm^3]$;

$V_{SEM\ picture} = volume\ above\ Si - waver\ adapted\ to\ chosen\ magnification\ [\mu L]$; $a\ \&\ b = length\ \&\ width\ of\ the\ SEM\ image\ (as\ an\ example\ for\ magnification\ factor\ 300x)\ [\mu m]$;

$PNC = particle\ number\ concentration\ \left[\frac{NP}{mL}\right]$; $N_{median} = number\ of\ particles\ in\ the\ SEM\ image\ [NP]$;

$DF = dilution\ factor$

Equation S2. Calculation of the limit of detection for particle masses (LOD$_{mass}$).

$$Limit\ of\ detection\ (LOD)_{mass}\ [fg] = \frac{(Corrected\ background - b) \cdot t_{dwell} \cdot \dot{V} \cdot \eta \cdot 10^9 \left[\frac{fg}{\mu g}\right]}{m \cdot f_d}$$

Legend

$b = intercept\ (ionic\ calibration\ curve)\ [CPS]$; $t_{dwell} = dwell\ time\ [0.0001\ s]$; $\dot{V} = sample\ inlet\ flow\ \left[\frac{L}{s}\right]$; $\eta = transport\ efficiency$; $m = slope\ (ionic\ calibration\ curve)$; $f_d = molar\ ratio\ (e.g.\ Si\ was\ measured\ but\ occurs\ as\ SiO_2) \rightarrow f_d = \frac{M(SiO_2)}{M(Si)}$; $PDT = Particle\ detection\ threshold$

$Corrected\ background\ (Gaussian)\ [CPS]: PDT - mean_{ionic\ background}$

$Corrected\ background\ (Poisson)\ [CPS]: PDT\ (here\ called\ limit\ of\ detection) - limit\ of\ criticality$[3]

Equation S3. Calculation of the limit of detection for particle sizes (LOD$_{size}$).

$$Limit\ of\ detection\ (LOD)_{size}[nm] = \sqrt[3]{\frac{6 \cdot 10^{21} \left[\frac{nm^3}{cm^3}\right] \cdot LOD_{mass}\ [fg]}{\pi \cdot 10^{15} \left[\frac{fg}{g}\right] \cdot \rho}}$$

Legend

$\rho = particle\ density \left[\frac{g}{cm^3}\right]$

REFERENCES

(1) Zernig, G.; Saria, A.; Kurz, M.; O'Malley, S. Physicochemical Properties of Ethanol. In *Handbook of Alcoholism*; CRC Press, 2000; pp 417–418. https://doi.org/10.1201/9781420036961-49.

(2) Longwic, R.; Sander, P.; Jańczuk, B.; Zdziennicka, A.; Szymczyk, K. Modification of Canola Oil Physicochemical Properties by Hexane and Ethanol with Regards of Its Application in Diesel Engine. *Energies* **2021**, *14* (15), 4469. https://doi.org/10.3390/en14154469.

(3) Lockwood, T. E.; Gonzalez De Vega, R.; Clases, D. An Interactive Python-Based Data Processing Platform for Single Particle and Single Cell ICP-MS. *J. Anal. At. Spectrom.* **2021**, *36*, 2536–2544. https://doi.org/10.1039/d1ja00297j.
